# Functional Brain Networks in Mild Cognitive Impairment Based on Resting Electroencephalography Signals

**DOI:** 10.3389/fncom.2021.698386

**Published:** 2021-10-20

**Authors:** Nadia Youssef, Shasha Xiao, Meng Liu, Haipeng Lian, Renren Li, Xi Chen, Wei Zhang, Xiaoran Zheng, Yunxia Li, Yingjie Li

**Affiliations:** ^1^School of Communication and Information Engineering, Shanghai Institute for Advanced Communication and Data Science, Shanghai University, Shanghai, China; ^2^Department of Neurology, Tongji Hospital, School of Medicine, Tongji University, Shanghai, China; ^3^School of Life Sciences, Shanghai Institute for Advanced Communication and Data Science, Shanghai University, Shanghai, China

**Keywords:** connectivity, graph theory, resting EEG, mild cognitive impairment, minimum spanning tree, machine learning

## Abstract

The oscillatory patterns of electroencephalography (EEG), during resting states, are informative and helpful in understanding the functional states of brain network and their contribution to behavioral performances. The aim of this study is to characterize the functional brain network alterations in patients with amnestic mild cognitive impairment (aMCI). To this end, rsEEG signals were recorded before and after a cognitive task. Functional connectivity metrics were calculated using debiased weighted phase lag index (DWPLI). Topological features of the functional connectivity network were analyzed using both the classical graph approach and minimum spanning tree (MST) algorithm. Subsequently, the network and connectivity values together with Mini-Mental State Examination cognitive test were used as features to classify the participants. Results showed that: (1) across the pre-task condition, in the theta band, the aMCI group had a significantly lower global mean DWPLI than the control group; the functional connectivity patterns were different in the left hemisphere between two groups; the aMCI group showed significantly higher average clustering coefficient and the remarkably lower global efficiency than the control. (2) Analysis of graph measures under post-task resting state, unveiled that for the percentage change of post-task vs. pre-task in beta EEG, a significant increase in tree hierarchy was observed in aMCI group (2.41%) than in normal control (−3.89%); (3) Furthermore, the classification analysis of combined measures of functional connectivity, brain topology, and MMSE test showed improved accuracy compared to the single method, for which the connectivity patterns and graph metrics were used as separate inputs. The classification accuracy obtained for the case of post-task resting state was 87.2%, while the one achieved under pre-task resting state was found to be 77.7%. Therefore, the functional network alterations in aMCI patients were more prominent during the post-task resting state. This study suggests that the disintegration observed in MCI functional network during the resting states, preceding and following a task, might be possible biomarkers of cognitive dysfunction in aMCI patients.

## Introduction

Mild cognitive impairment (MCI) refers to a clinical condition that reflects an intermediate mental state between normal cognition and dementia (Petersen et al., 1995). In particular, it is a disorder characterized by a cognitive decline affecting elderly individuals, without meeting the condition of dementia. Even if the MCI condition may remain stable over time, it could be a major risk factor for the conversion to Alzheimer’s disease (AD). Furthermore, structural and functional changes have been observed in the MCI group, suggesting that the dysfunction of brain networks starts during the MCI phase of AD (Wang et al., 2012). Accordingly, the investigation of the brain network characteristics of MCI might be a crucial step toward the diagnosis and medical treatment of dementia. In this connection, the synchronization of rhythmic activity of the cortical neuron, occurring at the scalp level, was shown in Rodriguez et al. (1999) to play an important role in extracting the global and local properties of brain connectivity in MCI and AD, such as electroencephalogram (EEG), which reflects the pattern of the temporal interdependence, or information flow dynamics of anatomically separated brain regions (Rodriguez et al., 1999). Brain changes in MCI and AD during resting state were addressed, using several approaches best exemplified by phase synchronization, coherence, Granger Causality, and phase slope index. In particular, the existing EEG studies pointed out that AD patients have altered functional connections between brain regions in resting condition with respect to healthy controls (Jeong et al., 2001; Babiloni et al., 2016; Başar et al., 2017). Specifically, Jeong et al. (2001) study observed an overall decrease in connectivity values within frontal and anterior-temporal regions. Particularly, amnestic mild cognitive impairment (aMCI), a dominant subtype of MCI, has a higher risk of conversion to dementia compared to the other cases of MCI. Functional connectivity in aMCI condition was investigated by many studies (Wang et al., 2013; Tóth et al., 2014; Zeng et al., 2015). Abnormalities of connectivity between brain areas seem to be a key characteristic between brain areas underlying memory deficits in aMCI, which has a high conversion rate to AD. For instance, the analysis of EEG functional connectivity through phase lag index (PLI), revealed a significant reduction of synchronization in delta and theta between the frontal and temporal, and frontal and parietal brain areas (Tóth et al., 2014). In the study conducted by Zeng et al. (2015), reduced synchronization was observed in the aMCI group in lower alpha, upper alpha, and beta bands. In the same study, the reported impaired connections appeared on the left hemisphere. The use of coherence for the study of functional connectivity in aMCI in Bian et al. (2014) study, showed decreased values at the theta band in left central-right central and left posterior-right posterior brain areas. Decreased coherence was also found in the alpha band in posterior, fronto-right temporal, fronto-posterior, right temporo-posterior regions (Bian et al., 2014). The application of the weighted phase lag index (WPLI) in Surya and Puthankattil work failed to detect significant variation in any of the patient-control groups (Das and Puthankattil, 2020). The absence of differences in the study (Das and Puthankattil, 2020), could relate to several factors: The sample size was relatively small (13 MCI, 20 Normal), and the subjects were only mildly affected. Considering the advantages of debiased weighted phase lag index (DWPLI) in mitigating the effects of volume conduction and the common reference problems compared to other measures of phase synchronization, it is still unknown how effective it would be in detecting brain functional changes in aMCI during resting state.

Together with functional connectivity, graph theory would provide a powerful framework for deeper insights into the organization of the brain network. It enables the characterization of brain network topology where nodes describe brain regions and links represent the pair-wise connectivity values (Rubinov and Sporns, 2010). Within this context, previous studies demonstrated that dementia is associated with the loss of small-world architecture, which is an indicator of the balance between the local connectedness and the global integration of a network (Tijms et al., 2013; Zeng et al., 2015; Zhang et al., 2015; Rossini et al., 2020). However, the findings in regard to network topology in MCI have not always been consistent. In fact, some results have been shown to reveal the lack of significant changes of network topology in MCI when compared to the healthy group, while others demonstrated decreased or increased “small-worldness” (Seo et al., 2013; Vecchio et al., 2014; Zeng et al., 2015; Zhang et al., 2015). For example, Vecchio et al. (2014) reported a significant increase in the clustering coefficient for the MCI group, without any change in the path length of the network. In contrary, Seo et al. (2013) reported a decrease in local clustering of networks in MCI compared to normal cognitive subjects. The discrepancies in those outcomes may be due to methodological issues such as thresholding. This operation makes, in fact, the comparison between networks of different sizes and different edge densities challenging, and leads to contradictory results. A possible alternative to avoid those limitations is the use of the minimum spanning tree (MST) (Stam et al., 2014). The MST is a sub-graph that represents the backbone of the original network, containing unique connections between nodes without cycles (Stam et al., 2014). The potential of MST is in avoiding the random choices of thresholds and eliminating the bias that originates from comparing network characteristics using traditional approaches.

It should be highlighted that up till now most EEG studies examining functional networks in MCI focused on pre-task resting state and it is still unclear how connectivity patterns vary during a post-task resting state. The post-task resting state networks were, generally, viewed as a “task-driven” functional network state associated with cognition and they were affected by preceding tasks (Grigg and Grady, 2010; Wang et al., 2012). However, it is still not much known about the abnormalities that could be observed in brain functional networks during post-task resting state, under dementia condition. Therefore, the investigation of EEG data under resting condition following a cognitive task could potentially provide new information about the alteration of brain network organization for aMCI patients. This will be the main topic of the present study. Specifically, the current study is aimed to look into functional connectivity in the pre-/post EEG resting signals and to use a machine learning algorithm to assess which condition gives better performance in terms of the classification of aMCI vs. normals. It is anticipated that there would be a reduction in functional connectivity of aMCI when compared to the case of the healthy group. It is also hypothesized that functional network changes in aMCI may be revealed following emotion regulation. Considering the relationship between cognition and cognitive reappraisal (Xiao et al., 2021), one of the most effective emotion regulation strategies, cognitive reappraisal was chosen in this study. To this end, DWPLI, a method that is insensitive to volume conduction, was applied between all channels of interest for EEG signals in different bands. The DWPLI based connectivity values were averaged for intra- and inter-hemispheric channel pairs to explore abnormality of the functional connectivity. Following this, brain graph metrics were derived from the weighted connectivity matrices using MST and classical graph measures. We expect that the combination of DWPLI, MST, and cognitive status of participants would give more insights into the discriminative features of aMCI. Therefore, classification was applied using random forest for the purpose of assessing the best predictors of aMCI status under resting conditions.

## Materials and Methods

### Participants

The study sample consisted of 94 individuals, who were recruited from the neurology department of Tongji Hospital in Shanghai. Forty-three of them were diagnosed as amnestic mild cognitive impaired (aMCI group), while 51 showed no sign of cognitive impairment. All participants underwent a comprehensive Neuropsychological Test Battery (NTB) that included the Mini-Mental State Examination (MMSE), the Clinical Dementia Rating scale (CDR), and tests that evaluate the memory function, language function, executive function, and spatial visual navigation function (Xiao et al., 2021; see Supplementary Materials for more details). The neuro-psychological indices of participants are given in Table 1. As can be noted from this table, a significant difference is obtained for MMSE score. Moreover, there are no relevant dissimilarities in gender, education status, and age. The subjects taking medicines (antidepressants, cholinesterase inhibitors, and hypnotics) with dementia, mental illness, history of stroke, or Parkinson’s disease were also excluded (see Supplementary Materials for the details of the exclusion criteria).

**TABLE 1:** Clinical characteristics between amnestic cognitive impaired and control subjects.

	aMCI (*N* = 43)	Controls (*N* = 51)	*p*-value
**Gender (M/F)**	17/26	26/25	0.303
**MMSE**	23.86 ± 3.3202	27.451 ± 1.7241	*p* < 0.001
**Age (50∼92)**	69.465 ± 8.9824	69.020 ± 7.3525	0.764
**Education (years)**	10.28 ± 3.202	11.10 ± 2.795	0.159

*Data are presented as mean ± SD. p-values of gender was obtained by chi-square test; p-values of medication was obtained by Fisher’s Exact Test, p-values for comparison in other demographic data, neuropsychological performance was acquired by independent sample t-test. SD, standard deviation; MMSE, Mini-Mental State Examination; M, male; F, female.*

The aMCI patients were selected using the following three sets of inclusion (Petersen, 2004): (1) had complaint of memory/cognitive deficit by subject and his caregiver; (2) CDR = 0.5; (3) When any of the following two criteria were met: (a) At least two tests within the memory cognitive domain scored below the established cut off; (b) At least two cognitive domains (memory and other cognitive domain) were in disrupted. Besides, the screening of controls was chosen according to the following inclusion criteria (1) Without complaint of memory or cognitive deficit (2) MMSE > 20 for primary school or above, MMSE > 24 for junior high school or above; (3) be capable of performing daily errands; (4) all cognitive domain were all within the normal range; (5) no history of diabetes.

Written informed consents were obtained from all participants. Our study was approved by the Ethics Committee for Clinical Research in Tongji Hospital.

### Electroencephalography Protocols

Five-minute scalp EEG data were recorded from 64 scalp electrodes positioned based on the international extended 10-10 system using a NeuroScan SynAmps2 (SynAmps2 Model 8050 EEG amplifier and data acquisition system, Abbotsford, Victoria, Australia) during an eye-closed resting-condition prior (Rest 0) and after cognitive reappraisal task (Rest 1) (see Supplementary Figure 1).

All electrodes were referenced to a central electrode placed between Cz and CPz, and grounded to AFz. The impedance between channels was maintained below 20 kΩ.

EEG oscillations were recorded at a sampling frequency of 1,000 Hz, and then band-pass filtered between 0.1 and 95 Hz. Line noise of 50 and 100 Hz were filtered out using a notch filter. Independent component analysis (ICA) was then applied to minimize the presence of oculographic, cardiographic, and myographic artifacts. The cleaned data were referenced to an infinite point using REST software (Yao, 2001). Afterward, EEG data were segmented into 4-s artifact-free epochs in all subjects. The artifact-free EEG segments were exported for functional connectivity analysis in the following band frequencies; delta (1–4 Hz), theta (4–7 Hz), low alpha (8–10 Hz), upper alpha (10–13 Hz), beta (13–30 Hz), and gamma (30–45 Hz). For further analysis, the connectivity strength was averaged over the following regions of interests (see also Supplementary Figure 2): (1) left prefrontal lobe (LF) (F7, F3, FP1); (2) right prefrontal lobe (RF) (F8, F4, FP2); (3) left temporal lobe (LT) (FT7, T7, TP7); (4) right temporal lobe (RT) (FT8, T8, TP8); (5) left parietal lobe (LP) (CP3, P7, P3); (6) right parietal lobe (RP) (CP4, P4, P8); (7) left center (LC) (FC3, C3); (8) right center (RC) (FC4, C4); (9) medial parietal (MP) (P1,P2, Pz, POz); (10) occipital (O) (O1,O2,Oz). Subsequently, graph analysis was performed using the classical graph approach and MST. The aforementioned processing procedure was performed using the custom-made scripts and open-source toolboxes in MATLAB, including: (1) EEGLAB MATLAB (Delorme and Makeig, 2004) for data pre-processing, (2) Fieldtrip for connectivity analysis (Oostenveld et al., 2011), and (3) Brain Connectivity Toolbox, BCT for graph theoretical analysis (Rubinov and Sporns, 2010).

### Debiased Weighted Phase Lag Index

We used the DWPLI as a measure of functional connectivity. The weighted phase-lag index (WPLI) is an extension of the phase-lag index (PLI) (Vinck et al., 2011). PLI estimates the asymmetry in the instantaneous phase distribution between two oscillations. It measures the phase synchronization depended on the distribution of instantaneous phase difference between two signals. The weighted version of PLI is calculated by weighing the contribution of phase leads and lags using the magnitude of the imaginary part of the cross-spectrum between the two signals. The weighted version of PLI is defined as the fraction of weighted phase lags by the magnitude of the imaginary part of the complex cross-spectrum. It aims to separate amplitude and phase-synchronous based artifacts that are mixed with brain activity.


(1) WPLI=x⁢y1n⁢∑i=1n|i⁢m⁢a⁢g⁢(Sx⁢y⁢t)|⁢s⁢g⁢n⁢(i⁢m⁢a⁢g⁢(Sx⁢y⁢t))1n⁢∑i=1n|i⁢m⁢a⁢g⁢(Sx⁢y⁢t)|

Here *S*_*xyt*_ refers to the cross-spectral density between x and y time series data at time point t in the complex plane xy. Sgn is the sign function (−1, +1 or 0). DWPLI is a debiased estimator of squared WPLI, has been shown to be immune to volume conduction effects by excluding the connectivity zero-lag connectivity generated from the mixture of real and spurious relationships. By calculating the DWPLI values between all pairs of electrodes, we obtained a 60 × 60 adjacency matrix for five frequency bands, which we employed for the network analyses (see below).

### Graph Theoretical Analysis

In our study, we used the graph theory combined with DWPLI for constructing the brain network. This network is represented by both vertices and edges (Rubinov and Sporns, 2010). The vertices refer to electrodes. Edges between pairs of nodes were quantified by DWPLI values. The brain networks were constructed by applying a proportional threshold, which selects 10% of the strongest connections from DWPLI connectivity matrices. Next, the computation of graph metrics in terms of average node degree, normalized average clustering coefficient (CC), normalized characteristic path length, average local and global efficiency (GE), and small-world index, were extracted considering each subject separately. The determination of these metrics is subsequently explained. The degree of a node in a weighted undirected graph is used to refer to the number of links connected to the node. It provides us with a measure of a node’s hubness that can be computed as *K*_*i*_ ∑_*j*∈*N*_ *W*_*ij*_, where *W*_*ij*_ stands for the connection weight between node I and node j. Concerning the average clustering coefficient (CC), it is used to measure the network segregation based on the number of triangles in the network. The neighbors of a vertex are defined by the vertices that are directly connected to it. Locally, the clustering coefficient is equal to the fraction of the vertex neighbors that are also neighbors with each other (Rubinov and Sporns, 2010). CC quantifies the tendency of networks to form interconnected groups of brain regions (Rubinov and Sporns, 2010). It is defined as


(2) $$C_{i}=\frac{\sum_{k\neq i}\sum_{l\neq i,l\neq k}W_{i\,k}\,W_{i\,l}\,W_{k\,l}}{\sum_{k\neq i}\sum_{l\neq i,l\neq k}W_{i\,k}\,W_{i\,l}}$$

where *W*_*ij*_ is the edge weight between node i and node j, derived from the connectivity matrices, which is estimated using the Fieldtrip toolbox (Oostenveld et al., 2011). Then, the average of all the local clustering coefficients can be computed as


(3) $$C=\frac{1}{N}\,\sum_{i=1}^{N}C_{i}$$

Regarding the local efficiency, it has a similar role to the clustering coefficient. It quantifies the efficiency of information transmission within local regions and reveals how efficient the communication of a node i is with its neighbors when node i is removed. It is computed by means of the following expression


(4) $$L\,E=\frac{1}{2\,N}\,\sum_{i\in N}\frac{\sum_{j,h\in N,j\neq i}{\left(W_{i\,j}\,W_{i\,h}\,\left[{\left(d_{j\,h}\,\left(N_{i}\right)\right)}^{-1}\right]\right)}^{1/3}}{\left(K_{i}\right)\,\left(K_{i}-1\right)}$$

where *d*_*jh*_ denotes the shortest path length between nodes i and j, while *N*_*i*_ is the number of nodes in the network.

As for the characteristic path length, it is the most common measure of functional integration, which estimates the potential path of communication between brain regions. It is the average number of edges along the shortest path that connects one node to another. It is obtained as the average of the shortest path lengths between all possible pairs of nodes in a network


(5) $$L=\frac{1}{n}\,\sum_{i\in N}\frac{\sum_{j\in N,j\neq i}d_{i\,j}}{n-1}.$$

As for the global efficiency, it is measured by finding the average inverse of the shortest path according to


(6) $$G\,E=\frac{1}{N}\,\sum_{i\in N}\sum_{j\in N,i\neq j}\frac{{\left(d_{i\,j}\right)}^{-1}}{N-1}$$

The small-world index is a measure of small-worldness and it is referred to as the outcome of the comparison of the properties of the network with respect to corresponding random networks. It is defined as σ = γ/λ, where γ = C/C_*random*_ and λ = L/L_*random*_. C_*random*_ and L_*random*_ denote the values of the clustering coefficient and path characteristic of the matched random reference graphs, respectively. The small-world architecture has the advantages of both random and regular networks. It is characterized by a high-clustering coefficient similar to that of regular networks, and by short characteristic path lengths close to those of pure random graphs (Watts and Strogatz, 1998). The randomized networks were obtained by reshuffling the original values of connectivity matrices. In our case, 50 random networks were generated, and for which λ and γ were calculated for both aMCI and control groups. Brain networks, with small-world property greater than 1 (i.e., σ > 1), are known to reflect better information processing performance with less disruption (Stam and Reijneveld, 2007).

### Minimum Spanning Tree

The second and alternative technique we have used for describing the brain network is the MST. The MST approach gives a subgraph derived from the connectivity matrix, where all nodes of the network are connected but without forming loops. For n nodes in the network, the MST algorithm produced a tree with n-1 strongest links, employing Kruskal’s algorithm (Kruskal, 1956). The MST-related characteristics parameters analyzed in the current study include maximum degree (K_*max*_), betweenness centrality (BC), eccentricity (ECC), diameter (D), leaf fraction (Lf), and tree hierarchy (Th). The MST can be viewed as the backbone of the brain network, and changes in the topological structure of MST are associated with change trends in the brain network. MST-based network, with a low diameter and high leaf number, tends to represent an optimal network, also known as a “star-shaped network.” Eccentricity determines the longest distance between a reference node and any other node. It has commonly been interpreted as the efficient information flow in the network observed from the least central node. Betweenness centrality is an indicator of network centrality in a network. The degree, BC, and ECC characteristic parameters are useful in studying the nodal relevance in a network. Finally, tree hierarchy characterizes the hierarchical topology of a tree. Specifically, it captures the balance between diameter reduction and overload prevention resulting from a high BC. The definitions of MST properties are given in Supplementary Table 1).

### Machine Learning and Assessment Method

The Random Forest algorithm was used to classify normal vs. aMCI. This algorithm is a machine learning method based on decision trees (Breiman, 2001). It works by the construction of many decision tress, the random selection of subsets of features with replacement, and the evaluation of the best features based on majority voting. The predictions were made depending on the mentioned measures. Furthermore, this method has the advantage to combine decision trees with bagging allowing the decrease of over-fitting. Two sets of data describing the functional connectivity and topology measures were used individually as classification features. Then, we combined both sets with cognitive test (MMSE) to check the prediction performance of each model. We used recursive elimination for selecting features of the model. RFE is a feature selection method that fits a model and removes the weakest feature (or features) until the specified number of features is reached. Leave-one-out cross validation has been employed in our case since the sample size is relatively small. Finally, the performance was evaluated by considering the accuracy, sensitivity, as well as F1 score using a confusion matrix. It is a specific table layout useful for the visualization of the classification performance. The confusion matrix is composed of two rows and two columns reporting the number of true positives (TP), false positives (FP), true negatives (TN), and false negatives (FN). True (T) and false (F) stand for the predicted result, while positives (P) and negatives (N) indicate the actual condition (Tavares et al., 2019).

The evaluation of the results was conducted in terms of accuracy, precision, sensitivity, and F1 score. The accuracy is used for the calculation of the percentage of correct predictions out of the total samples. Along with the accuracy, the precision describes the rate of correct predictions among the predicted positive conditions. On the other side, sensitivity is a measure of the percentage of correct predictions among the real positive ones. Finally, the F1 is a balance for sensitivity and precision. These metrics are, respectively, obtained according to,


(7) $$A\,c\,c\,u\,r\,a\,c\,y=\frac{T\,P+T\,N}{T\,P+T\,N+F\,P+T\,N}$$

(8) $$P\,r\,e\,c\,i\,s\,i\,o\,n=\frac{T\,P}{T\,P+F\,P}$$

(9) $$S\,e\,n\,s\,i\,t\,i\,v\,i\,t\,y=\frac{T\,P}{T\,P+F\,P}$$

(10) $$F\,1=2^{*}\,\frac{P\,r\,e\,c\,i\,s\,i\,o\,n^{*}\,S\,e\,n\,s\,i\,t\,i\,v\,i\,t\,y}{P\,r\,e\,c\,i\,s\,i\,o\,n+S\,e\,n\,s\,i\,t\,i\,v\,i\,t\,y}$$

### Statistical Analysis

Demography and neuropsychological assessment data were assessed with the Shapiro Wilk test for normality. We compared the demographic and clinical measures between aMCI and controls using Mann-Whitney U and Chi-square tests. EEG measures were found to be non-normally distributed. We also used Fisher’s exact test to compare the number of individuals taking medicines (antidepressants, cholinesterase inhibitors, and hypnotics) between groups. Functional connectivity was averaged globally and regionally for each resting state, pre-and post-task, respectively, prior to the statistical analysis. The obtained values and graph metrics (MST and classical graph measures) were compared using Mann-Whitney *U*-test for each case. Finally, we used Mann-Whitney *U*-test to check the group differences in the change in percentage between pre-and post-task resting state given by [(post-pre)/pre]^∗^100.

*P*-values were adjusted for multiple comparison problem using with false discovery rate (FDR). The significance level (*p*-value) was set to 0.05. All the statistical analysis were carried out in Matlab (version 9.3, Mathworks, Natick, MA, United States) and SPSS for Windows (Version 23.0. Armonk, NY: IBM Corp.).

## Results

### Functional Connectivity

#### Functional Connectivity in Pre-task Resting State

**FIGURE 1:**
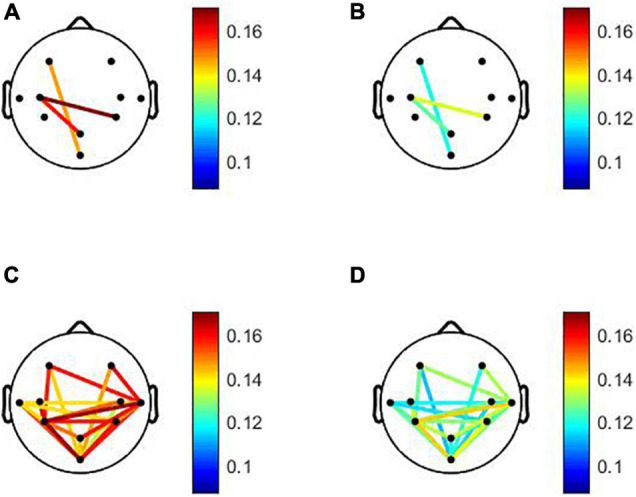
Statistically significant local connectivity patterns between two groups: Control group **(A)**. **(C)** vs. amnestic mild cognitive impairment (aMCI) group **(B)**. **(D)** Under pre- and post-resting states, respectively. Only statistically significant connectivity patterns. obtained by applying the false discovery rate corrected Mann-Whitney *U*-test (*p* < 0.05). Are displayed for each group using a color code: red color tones indicate higher connectivity level. Whereas blue color tones denote low values of connectivity. For the pre-task resting state. Under post-task resting state. The connections are characterized by an overall decrease in aMCI compared to the normal group. The low values of connectivity were observed between occipital and the other brain regions for aMCI and normal groups. For aMCI group. The minimum values were shown between occipital and left frontal brain areas. For normal group the minimum local connectivity was observed between occipital and right central areas. For aMCI and normal groups maximum local connectivity in the theta band was observed between left and right parietal regions.

The statistical evaluation of both global and local connectivity in the pre-task resting condition showed significant differences between aMCI and control groups in the theta band (*p* = 0.0044). No significant differences were found in other EEG bands. The results showed overall lower connectivity in the aMCI group than in the control group. The mean theta-band DWPLI was about 23% less in the aMCI group [0.0557 (95%CI = 0.0468, 0.0647)] as compared to the control group [0.0728 (95%CI = 0.0636, 0.082)]. Statistically significant differences between groups were found after the FDR correction between left frontal and occipital, between left central and medial parietal and between left central, and right parietal brain regions. Comparisons of the local connectivity between control and aMCI groups are presented in Table 2. Figure 1 shows the statistically significant connectivity patterns, obtained by applying the FDR-corrected Mann-Whitney *U*-test. The maximum connectivity was observed between the left central and right parietal for both groups. The lowest connectivity values were seen between the left frontal and occipital brain regions.

**TABLE 2:** DWPLI connectivity values between different brain regions.

Frequency band	Interregional connections	DWPLI*				*p*-value	Corrected *p*-value	Condition
					
		aMCI*		Control				
					
		Mean	*SD*	Mean	*SD*			
	LF O*	0.0949	0.0695	0.1364	0.0842	0.0027	0.0408	
Theta band	LC MP*	0.1059	0.0673	0.1524	0.0814	0.0027	0.0408	Pre-task
	LC RP*	0.1209	0.0843	0.1706	0.0876	0.0002	0.0408	
	LF O*	0.0870	0.0649	0.1291	0.0819	0.0036	0.0321	
	LF RT*	0.1129	0.0962	0.1537	0.0958	0.0060	0.0324	
	LF LP*	0.1066	0.0821	0.1507	0.1045	0.021	0.0495	
	RF O*	0.0980	0.0743	0.1366	0.0768	0.0025	0.0321	
	RF RT*	0.1149	0.0898	0.155	0.0959	0.0094	0.0324	
	LC LP*	0.1198	0.1033	0.1577	0.1059	0.0223	0.0495	
	RC O*	0.0897	0.0864	0.1163	0.0788	0.0092	0.0324	
	RC LP	0.096	0.0786	0.1294	0.0851	0.0198	0.0495	
	MP O	0.0963	0.0609	0.1290	0.0707	0.0077	0.0324	
	MP RT	0.1065	0.0904	0.1473	0.0776	0.0018	0.0321	
Theta band	MP LP	0.1099	0.0773	0.1503	0.0874	0.0077	0.0324	
	MP RP	0.1239	0.0930	0.1537	0.0825	0.0115	0.0345	
	O LT*	0.0933	0.068	0.1281	0.0723	0.0016	0.0321	Post-task
	O RT	0.1039	0.0954	0.1281	0.0756	0.0101	0.0324	
	O LP	0.0894	0.0522	0.1283	0.0722	0.0067	0.0324	
	O RP	0.0989	0.0701	0.1353	0.0819	0.0242	0.0495	
	LT RT	0.1105	0.0829	0.1526	0.0828	0.0029	0.0321	
	LT LP	0.1085	0.0875	0.1431	0.0816	0.0090	0.0324	
	LT RP	0.1183	0.0815	0.1527	0.0869	0.0175	0.0464	
	RT LP	0.1165	0.0973	0.1422	0.0731	0.0142	0.0400	
	RT RP	0.1293	0.1178	0.1668	0.0906	0.0081	0.0324	
	LP RP	0.1303	0.088	0.1668	0.0872	0.0242	0.0495	

*DWPLI, Debiased weighted phase lag index; aMCI, amnestic mild cognitive impairment; LF, left prefrontal; RF, right prefrontal lobe; LT, left temporal lobe; RT, right temporal lobe; LP, left parietal lobe; RP, right parietal lobe; LC, left center; RC, right center; MP, medial parietal; O, occipital.

#### Functional Connectivity in Post-task vs. Pre-task Resting State

During the post-task resting state, the DWPLI results revealed that EEG activity, in the aMCI group, is characterized by a global decrease in functional connectivity in the theta band with *p* = 0.0061 as reported in Table 3. The mean theta-band DWPLI was about 20% less in the aMCI group [0.057 (95%CI = 0.0457, 0.0684)] as compared to the control group [0.0713 (95%CI = 0.062, 0.08)]. Specifically, DWPLI values showed a reduction of local functional connectivity, in the theta band, between occipital and other brain regions, between temporal and parietal, and also between temporal and frontal brain areas, in both hemispheres. Results for inter-regional connections, considering aMCI and normal groups, are given in Table 2 and displayed in Figure 1. As shown in Figure 1, only statistically significant connectivity patterns, obtained by applying the FDR-corrected Mann-Whitney *U*-test, are displayed for each group using a color code: red color tones indicate higher connectivity level, whereas blue color tones denote low values of connectivity. As can be noted, the connections are characterized by an overall decrease in aMCI compared to the normal group. The low values of connectivity were observed between the occipital and the other brain regions for aMCI and normal groups. For aMCI group, the minimum values were shown between occipital and left frontal brain areas. For the normal group, the minimum local connectivity was observed between occipital and right central areas. For aMCI and normal groups, maximum local connectivity in the theta band was observed between left and right parietal regions (see Figure 1).

**TABLE 3:** Network parameters descriptive for both aMCI and control groups.

Frequency band	Graph index	Pre-task resting state					Post-task resting state				
			
		Controls (*N* = 51)		aMCI (*N* = 43)		*p*-value	Controls (*N* = 51)		aMCI (*N* = 43)		*p*-value
							
		Mean	*SD*	Mean	*SD*		Mean	*SD*	Mean	*SD*	
Delta	Mean DWPLI	0.0294	0.0247	0.024	0.0133	0.4255	0.0278	0.0149	0.0302	0.0192	0.7847
	Average CC	0.8427	0.2292	0.8151	0.2591	0.1918	0.7911	0.2827	0.8001	0.2759	0.9516
	Average LE	0.0586	0.0426	0.0468	0.0312	0.0996	0.0513	0.0309	0.055	0.0316	0.2581
	Global efficiency	0.1083	0.0807	0.0928	0.055	0.3705	0.1016	0.0517	0.1106	0.06	0.485
	Path characteristic	1.0711	0.0808	1.0745	0.0763	0.5337	1.0713	0.0835	1.0594	0.0679	0.5387
	Small random index	0.7857	0.1991	0.7581	0.2271	0.1841	0.7377	0.2557	0.7575	0.2628	0.7499
	Leaf fraction	0.4606	0.0453	0.4493	0.0542	0.2637	0.4749	0.0512	0.4659	0.0596	0.3282
	Between centrality	0.6539	0.0494	0.6579	0.0522	0.9033	0.6648	0.0559	0.6604	0.0583	0.7413
	Diameter	0.2832	0.0431	0.2988	0.0574	0.2072	0.2832	0.0501	0.2913	0.0491	0.231
	Max degree	0.099	0.0215	0.1004	0.0237	0.8108	0.1062	0.0305	0.1093	0.0266	0.2686
	Eccentricity	0.22	0.0322	0.2304	0.0406	0.1983	0.2202	0.0385	0.227	0.0388	0.2217
	Tree hierarchy	0.354	0.0428	0.344	0.0524	0.288	0.359	0.0434	0.3546	0.0492	0.5848
Theta	Mean DWPLI	0.0728	0.0327	0.0557	0.029	**0.0044**	0.0713	0.0326	0.057	0.037	**0.0061**
	Average CC	0.7408	0.241	0.8827	0.3455	**0.0362**	0.7819	0.2422	0.7849	0.2768	0.9516
	Average LE	0.1106	0.0406	0.1108	0.0429	0.9939	0.1211	0.0377	0.0993	0.0527	**0.0105**
	Global efficiency	0.2146	0.0707	0.1811	0.0701	**0.0128**	0.2163	0.0703	0.1809	0.0878	**0.0097**
	Path characteritstic	1.0874	0.062	1.1064	0.0989	0.6819	1.0886	0.0915	1.116	0.128	0.2518
	Small random index	0.682	0.2149	0.7974	0.3013	0.0511	0.715	0.2019	0.7127	0.2687	0.7499
	Leaf fraction	0.5028	0.0584	0.5002	0.0718	0.6335	0.4882	0.0663	0.4864	0.073	0.9454
	Between centrality	0.6719	0.0542	0.6668	0.0575	0.3091	0.6574	0.0498	0.6604	0.0584	0.9667
	Diameter	0.2655	0.042	0.2767	0.0462	0.0675	0.2805	0.0465	0.2944	0.0549	0.1712
	Max degree	0.1196	0.0355	0.1202	0.0377	0.9261	0.1186	0.0464	0.114	0.0392	0.6482
	Eccentricity	0.2071	0.032	0.2133	0.034	0.1515	0.2166	0.0351	0.2277	0.0414	0.1637
	Tree hierarchy	0.376	0.0499	0.3759	0.05	0.8465	0.3724	0.0508	0.3692	0.052	0.9879
Low alpha	Mean DWPLI	0.1646	0.0885	0.1336	0.0765	0.095	0.168	0.084	0.1471	0.0833	0.2161
	Average CC	0.7224	0.2258	0.7592	0.2285	0.3585	0.731	0.2257	0.716	0.2249	0.5952
	Average LE	0.2311	0.0805	0.2161	0.085	0.3995	0.254	0.0857	0.2335	0.0834	0.2812
	Global efficiency	0.4202	0.1369	0.3744	0.1289	0.1093	0.4331	0.1298	0.3953	0.1255	0.1329
	Path characteritstic	1.0561	0.0522	1.0729	0.0763	0.3995	1.0604	0.0731	1.0748	0.0657	0.1649
	Small random index	0.6806	0.1933	0.7026	0.1831	0.4616	0.691	0.2126	0.6676	0.2133	0.3911
	Leaf fraction	0.4915	0.053	0.4907	0.055	0.9666	0.4832	0.047	0.4955	0.0552	0.2654
	Between centrality	0.6675	0.0585	0.6532	0.0493	0.2811	0.6781	0.0594	0.6576	0.0446	0.1135
	Diameter	0.2798	0.0454	0.283	0.0391	0.4479	0.2745	0.0385	0.2893	0.0612	0.4069
	Max degree	0.118	0.0398	0.1202	0.035	0.4565	0.1167	0.0307	0.119	0.0318	0.7038
	Eccentricity	0.2166	0.0331	0.2202	0.0285	0.3164	0.2114	0.0287	0.2233	0.045	0.4038
	Tree hierarchy	0.3712	0.0549	0.3772	0.0473	0.3932	0.359	0.0463	0.3776	0.043	0.0727
Upper alpha	Mean DWPLI	0.136	0.079	0.1167	0.0656	0.2613	0.1304	0.0762	0.1284	0.0764	0.9094
	Average CC	0.7721	0.2146	0.8036	0.2814	0.559	0.8309	0.2365	0.7735	0.2292	0.4081
	Average LE	0.2019	0.0777	0.1901	0.0742	0.4803	0.1993	0.0777	0.1929	0.0837	0.6875
	Global efficiency	0.3616	0.1348	0.3274	0.1255	0.1892	0.3431	0.1299	0.3441	0.1365	0.9335
	Path characteritstic	1.0853	0.0814	1.1037	0.0816	0.1472	1.0816	0.062	1.0943	0.1362	0.6931
	Small random index	0.7097	0.1816	0.727	0.2385	0.7499	0.7657	0.2017	0.7112	0.2116	0.2846
	Leaf fraction	0.5028	0.0606	0.4903	0.0597	0.3603	0.4962	0.0576	0.4943	0.0526	0.9391
	Between centrality	0.6879	0.068	0.6765	0.0779	0.2261	0.6612	0.0627	0.6705	0.0553	0.2364
	Diameter	0.2672	0.0608	0.2822	0.0589	0.1267	0.2735	0.0419	0.2751	0.0381	0.7796
	Max degree	0.1261	0.0445	0.1326	0.0593	0.954	0.1212	0.045	0.1178	0.0348	0.9074
	Eccentricity	0.2076	0.0458	0.2178	0.0452	0.1931	0.2125	0.0308	0.2128	0.0285	0.9939
	Tree hierarchy	0.368	0.0506	0.3657	0.0526	0.814	0.3783	0.056	0.3719	0.0565	0.7043
Beta	Mean DWPLI	0.0473	0.024	0.0436	0.0216	0.4211	0.0474	0.0256	0.0499	0.0234	0.4898
	Average CC	1.0777	0.3949	1.0609	0.3593	0.8436	1.1639	0.3904	1.1011	0.3985	0.4850
	Average LE	0.0952	0.0363	0.0973	0.0374	0.9516	0.1057	0.0402	0.1108	0.0411	0.5796
	Global efficiency	0.1493	0.0652	0.1439	0.0605	0.7213	0.1481	0.065	0.1587	0.066	0.3869
	Path characteritstic	1.1424	0.0973	1.1327	0.1159	0.514	1.1448	0.1076	1.1355	0.0993	0.5337
	Small random index	0.934	0.3024	0.9323	0.2794	0.9576	1.0164	0.3201	0.9621	0.3118	0.4994
	Leaf fraction	0.5055	0.0688	0.4951	0.0500	0.3048	0.4922	0.0642	0.5022	0.0691	0.4715
	Between centrality	0.6737	0.0604	0.6677	0.0516	0.7528	0.6814	0.0621	0.6606	0.0494	0.1707
	Diameter	0.2742	0.0491	0.2763	0.0494	0.927	0.2689	0.0555	0.2763	0.0489	0.3499
	Max degree	0.1157	0.0301	0.1151	0.0333	0.7681	0.1186	0.0437	0.1163	0.0334	0.8148
	Eccentricity	0.2117	0.0367	0.2162	0.0372	0.6407	0.2102	0.0422	0.2157	0.0352	0.2812
	Tree hierarchy	0.377	0.0536	0.3718	0.0381	0.3911	0.3625	0.0453	0.3808	0.0493	0.0927
Gamma	Mean DWPLI	0.0529	0.0325	0.0519	0.0378	0.59	0.0531	0.0273	0.0588	0.0398	0.9939
	Average CC	1.0053	0.3864	1.0011	0.4833	0.6543	1.0453	0.4185	0.9632	0.405	0.3389
	Average LE	0.13	0.0631	0.1289	0.0644	0.6875	0.144	0.0628	0.1302	0.0678	0.2161
	Global efficiency	0.1869	0.0841	0.1928	0.1015	1	0.2059	0.0917	0.2146	0.1171	0.9033
	Path characteritstic	1.1215	0.1522	1.0772	0.1178	0.1719	1.1102	0.123	1.0966	0.148	0.457
	Small random index	0.9007	0.3178	0.9184	0.393	0.8794	0.9342	0.3343	0.8811	0.3491	0.4756
	Leaf fraction	0.4613	0.0529	0.4635	0.0561	0.9514	0.4629	0.0547	0.4643	0.0455	0.8127
	Between centrality	0.6467	0.0513	0.6514	0.0435	0.4922	0.6544	0.0539	0.6565	0.0434	0.4501
	Diameter	0.2954	0.0483	0.2893	0.0441	0.634	0.2978	0.0411	0.2846	0.0371	0.7845
	Max degree	0.1036	0.0241	0.1012	0.0225	0.633	0.1042	0.03	0.1019	0.0247	0.1399
	Eccentricity	0.2284	0.0348	0.2237	0.0317	0.6138	0.2322	0.0303	0.2226	0.0267	0.106
	Tree hierarchy	0.3585	0.0475	0.3569	0.0456	0.7789	0.3555	0.0482	0.3554	0.044	0.8555

*SD, standard deviation; Bold, significant difference between the two groups; DWPLI, Debiased weighted phase lag index; aMCI, amnestic mild cognitive impairment; LE, local efficiency; MMSE, Mini-Mental State Examination.*

The comparison of functional connectivity taken in percentage change [(post-pre)/pre]^∗^100 between control and aMCI groups using the Mann-Whitney *U*-test failed to capture significant changes between groups.

### Graph Analysis

#### Graph Analysis in Pre-task Resting State

Traditional graph and MST metrics were derived from DWPLI connectivity matrix for both groups in each frequency band. The outcome of the comparison of network characteristics, between the aMCI and normal groups, was reported in Table 3. Notable statistical differences were found for the global efficiency and normalized average clustering coefficient in the theta band. The aMCI group was characterized by a significantly higher normalized average clustering coefficient and higher global efficiency. The rest of the graph parameters (average local efficiency, normalized path characteristic, small-world index, between centrality, mean degree, eccentricity, leaf fraction, tree hierarchy) obtained during the pre-task resting state, do not differ significantly.

#### Graph Analysis in Post-task vs. Pre-task Resting State

During post-task resting state, it was observed that significant differences were only present in traditional graph metrics in the theta band, whereas MST metrics did not capture any dissimilarity between the two groups. Specifically, the aMCI group was characterized by a significant decrease in average local and global efficiencies. However, no prominent difference was found in terms of normalized path characteristic and clustering coefficient.

The outcome of comparison of graph measures taken in percentage change [(post-pre)/pre]^∗^100 between control and aMCI groups using Mann-Whitney *U*-test, revealed significant differences in the tree hierarchy relative in the beta band (*p* = 0.0369). Specifically, we noted higher changes in the aMCI group (2.4018%) compared to the control group (−3.8862%).

### Classification

Classification of DWPLI connectivity and graph measures for both groups was carried out, separately and also in combination, using the random forest algorithm. The classification results were reported in Table 4. The accuracy values obtained for graph metrics, after cross-validation, were lower than those obtained for DWPLI, which in its turn were shown to be lower than those obtained in the case of combined measures. Additionally, the classification performance in the post-task was higher than that corresponding to the pre-task case. Overall, the classification results shown in Table 4, considering the case of the combined measures, achieved the accuracy of 77.7 and 87.2% for the pre-and post-task, respectively (*p* = 0.0213). Measures of connectivity measured during the Rest0, regional connectivity between left central and other brain regions, between left and right frontal, and between right frontal and central brain areas, were relevant to the classification. Measures global efficiency, average clustering coefficient, and between centrality contributed the most to the classification model. For Rest1, connectivity between right central and occipital, between left frontal and right temporal, between left frontal and right central brain areas are among the top features for DWPLI connectivity, while global efficiency, local efficiency, and eccentricity, are the graph features that contribute most to our model.

**TABLE 4:** Classification results for pre- and post-task resting states.

	Accuracy	Precision	Sensitivity	F1 score	Condition
**Graph metrics**	0.649	0.661	0.725	0.692	
**DWPLI connectivity**	0.670	0.685	0.725	0.705	Pre-task
Combined measures*	0.777	0.800	0.784	0.792	
**Graph metrics**	0.681	0.691	0.745	0.717	Post-task
**DWPLI connectivity**	0.713	0.772	0.765	0.743	
Combined measures*	0.872	0.882	0.882	0.882	

*Combined measures comprise graph metrics. Debiased weighted phase lag index (DWPLI) connectivity and refers to Mini-Mental State Examination (MMSE) metric.

## Discussion

In our study, we attempted to identify the subtle changes in brain network topology and functional connectivity during the resting activity before and after the cognitive reappraisal phase. We are able to distinguish differences in functional connectivity and topological metrics. Additionally, the combination of these metrics and MMSE can achieve satisfactory classification accuracy.

### Reduction in Functional Connectivity in Amnestic Mild Cognitive Impairment

Evidence for functional connectivity loss in aMCI, with respect to the control group, during resting state prior to a task, were reported in various previous studies (Gómez et al., 2009; Zeng et al., 2015; Handayani et al., 2018). For instance, the use of PLI during resting state in Zeng et al. (2015), showed a significant decrease in lower alpha, upper alpha, and beta bands at both short and long intra-hemispheric connections. Similarly, a MEG study, reported disturbed beta-band functional connectivity in MCI using coherence and synchronization likelihood (Gómez et al., 2009). Following the same trend, MCI patients demonstrated a lower phase synchronization using phase lock value in the beta band occurring in the left temporo-parietal-occipital area. It should be noted that the majority of EEG-based studies demonstrated a lower level of global synchronization in MCI when higher frequency bands were used (i.e., alpha and beta bands), which is contrary to our main finding. The differences can probably be due to the method used to calculate the functional connectivity, or the heterogeneity of MCI, or the combination of both factors. Particularly, in our current study, the analysis of local and global connectivity during pre-task resting state showed significant differences between aMCI and control groups appearing in the theta band. Similar results were achieved when comparing the same global and local features between groups during the post-task resting state. The observed changes in connectivity for the aMCI group under the two conditions were, mainly, associated with decreased values of local and global coupling patterns. Therefore, the reduction in connectivity in both pre-and post-task resting states supports the hypothesis that individuals with aMCI are in a state described as a disconnection syndrome.

The study of local functional connectivity in the pre-task resting state a showed decreased level of inter-regional connections in the aMCI group between left frontal and occipital, between left central and medial parietal, and between left central and right parietal brain areas. Those abnormal functional connections were mainly distributed in the left hemisphere. This finding is in line with previous EEG studies in which early changes in AD and aMCI were detected in the left hemisphere followed by right hemisphere areas (Sankari et al., 2011; Zeng et al., 2015). Moreover, the disturbances in local connectivity reported in our work were consistent with earlier resting-state aMCI studies in which patterns of abnormal connections were identified in the theta band, particularly, between frontal and parietal brain regions (Tóth et al., 2014), between left central-right central, and between left and right posterior brain areas (Bian et al., 2014).

The alteration in global functional connectivity during post-task resting state analysis was captured in aMCI group in the theta band. The theta band was usually associated with memory, attention and cognitive control processes (Hasselmo and Eichenbaum, 2005). Furthermore, memory-related theta oscillations are generated by numerous structures in frontal and medial temporal regions and they facilitate memory processes by theta’s role in integrating these structures into coherent neurocognitive (Klimesch, 1999; Von Stein and Sarnthein, 2000; Wang et al., 2010). Therefore, memory and attention deficits, related to MCI, are likely to be reflected in altered theta activity. In line with this speculation, Deiber et al. (2009) reported decreased induced frontal theta oscillatory activity during a working memory task as a result of an increasing deficit of attention function in the MCI group. Cummins et al. (2008) also found a reduced level in theta power of fronto-central regions under memory task in aMCI individuals. Given that cognitive reappraisal is a phenomenon closely associated with the degree of attention and the reappraisal of emotion stimuli as well as theta oscillations in cognitive processes (Hajcak et al., 2010), the present disruption of theta EEG oscillations following cognitive reappraisal was expected.

The further study of inter-regional connections under the same condition, revealed connectivity disruptions in the same band, mainly between the occipital and the other brain regions, between temporal and frontal, and finally between temporal and parietal brain areas. The differentiation of connection patterns involved in the post-task resting state is in line with typical clinical features of MCI reported recently. For instance, previous studies have associated MCI with atrophy in the medial temporal lobes (Fellgiebel et al., 2004; Karas et al., 2004; Winblad et al., 2004; Pennanen et al., 2005; Devanand et al., 2007) and reduced white matter integrity in the hippocampus and other temporal regions (Kantarci et al., 2005; Fellgiebel et al., 2006). These medial temporal regions are important for memory, cognitive function, as well as the generation of theta oscillations (Raghavachari et al., 2001). Moreover, cognitive reappraisal strategy involved frontal and parietal control regions to modulate emotional responses in the amygdala (Buhle et al., 2014).

It should be noted that the functional deficit in the aMCI group in terms of altered connections was more obvious after the task performance compared to the pre-task resting state. Hence, we can speculate that the connectivity disturbances following the task performance might not only reflect the cognitive deficits in the aMCI, but also considered the lingering effect of the task on the subsequent resting activity, which might result in a slow return to default state. This speculation is in agreement with what has been reported by previous studies (Grigg and Grady, 2010; Kavcic et al., 2021). Furthermore, this study attributed the source of the prominent decrease of EEG spectral power after cognitive engagement to several reasons ranging from continuous activation of task-based networks, slowed re-activation of resting state networks, or probably new cognitive engagement associated with self-assessment of past task performance (Kavcic et al., 2021). Further study is required to probe the origins of the variability in post-task resting state EEG data.

### Abnormal Network Characteristics of Amnestic Mild Cognitive Impairment

We investigated changes in the network organization of aMCI using an advanced graph theoretical approach. Variations at network level have been observed between group in both pre-and post-resting states.

The comparison of graph features between normal and aMCI groups during pre-task resting state revealed significant changes in both integration and segregation measures. Particularly, a decrease in global efficiency associated with an increase in clustering coefficient was found in aMCI networks in the theta band. This result is in agreement with (Vecchio et al., 2014) study reporting increased clustering coefficient in aMCI group and unmodified path characteristic across groups. Along with the same context, it is worth mentioning that similar alterations in AD patients were, also, noticed in previous works in comparison to the healthy group (Morabito et al., 2015; Kabbara et al., 2018; Franciotti et al., 2019). For instance, Franciotti et al. (2019) study reported lower global efficiency in patients than in the control group. Other EEG works reported reduced global efficiency and increased clustering coefficient during AD progression (Morabito et al., 2015; Kabbara et al., 2018). Given that the same alterations were evident in AD and aMCI patients, we suggest that the progression of AD from the prodromal stage to dementia begins with the disruption of integration and segregation network characteristics.

For the post-cognitive reappraisal case, the graph analysis demonstrated significantly different network parameters (GE, LE) for both cohorts. Particularly, the aMCI dataset has statistically significantly lower network efficiency in terms of GE and LE compared to the control group in the theta band. Specifically, decreased global efficiency has been associated with less efficient information exchange between brain regions. In addition, the dropping down of local efficiency implies reduced clustered connections between topologically nearby neighbors. The alteration of these properties is related to network disintegration in aMCI, which is consistent with prior work on aMCI and AD (Berlot et al., 2016; Wang et al., 2018; Franciotti et al., 2019). The network-based alterations occurring after cognitive reappraisal confirmed that aMCI were characterized by a reduction of their global and local performances quantified by global and mean local efficiencies.

In addition to traditional graph analysis, we used MST measures. In contrast to classical graph metrics, the comparative study between patient and control groups using MST, during pre-task states, failed to capture statistical differences between the two groups. Those results are inconsistent with the ones reported previously by literature. In Jacini et al. (2018) work no differences were found between aMCI and controls in terms of global topological derived from MST. However, significant increases in values of eccentricity and diameter in the aMCI group have been reported in Das and Puthankattil (2020) study. Similarly, in Požar et al. (2020) work, a disintegration of network topology in the MCI group was revealed by MST. This inconsistency may be due to a consequence of different progressive stages involved in AD and MCI, differences in the clinical features of participants, and the connectivity metric used during analysis.

The increase of tree hierarchy in percentage change in aMCI from pre- to post-task resting state is described by a shift toward more random and less ordered network configuration as a result of prior cognitive activity. Therefore, aMCI functional networks measured immediately post-task demonstrated alteration in network organization compared to pre-task with a shift toward a more random network resulted from the loss of neurons and connections and random outgrowth of new connections (Stam, 2014). Moreover, the finding indicates the similarities between the aMCI networks and random networks. The same trend has been reported by previous studies in the MCI group (Liu et al., 2012; Zeng et al., 2015; Yan et al., 2018). Meanwhile, a reduction of the tree hierarchy in percentage change was observed in the normal group. We inferred that the network topology in the normal group shifted toward a less random and more ordered network configuration after the task performance.

### Multimodal Classification

Classification analysis performed for each resting state has considered connectivity measures, graph metrics, and cognitive test MMSE as classification features. The use of the combination of all the aforementioned features revealed an improved accuracy rate compared to the single approach. In particular, MMSE and functional connections measures were found to be highly ranked as important variables for the classification of healthy from aMCI patients. Moreover, classification results in the post-cognitive reappraisal task achieved an accuracy of 87.2% which is significantly higher than that obtained during the pre-cognitive reappraisal task (77.7%). Therefore, we could assume that greater sensitivity to detect cognitive decline in aMCI, was evident in the functional changes following the task performance. Within the same context, similar trend was found in Li et al. (2020) and Požar et al. (2020). The combination of cognitive tests, functional connections, and MST metrics, during the pre-task resting state, indicated an improved accuracy (86.5%) compared with classification using the single approach (Požar et al., 2020). In another study using the same paradigm as our work, the classification results of post-cognitive reappraisal, using different machine learning algorithms, showed better performance than the case pre-cognitive reappraisal task (Li et al., 2020). Hence, combining measurements of graph topology, functional connectivity, and cognitive test during resting state after cognitive engagement may be a sensitive biomarker to identify aMCI from healthy individuals.

Several limitations of this study merit to be mentioned here, and require further investigations. Firstly, the application of sensor-space-based measurement may overestimate functional connectivity due to the effects of volume conduction coupled with the lack of information about locations of the brain sources of EEG oscillations. Source-space functional connectivity may offer a solution to overcome these limitations given that EEG signals are appropriately processed. Another limitation is that the DWPLI can only estimate the existence of connections between EEG electrodes. Since the directional relationship is necessary for the full characterization of information flow in MCI, measures of effective or causal connectivity, such as phase slope index and Granger Causality, might be used as an alternative to the functional connectivity measures. Finally, it is noteworthy that a larger sample size should be used in futures studies to prove the usefulness of our methodology as a diagnostic tool.

Our study aimed to identify changes in network properties that could characterize aMCI by analyzing EEG resting data. Disintegration is an indication of disconnection between different brain regions, and it was observed in the aMCI functional network during resting states before and after a task. Therefore, the “disconnection hypothesis” in aMCI and AD was proved to be valid in this study and was more pronounced during the resting state following cognitive reappraisal. These alterations were frequency-specific in the theta band, which was classically associated with memory and attention processes. Furthermore, the combination of brain network characteristics and MMSE, computed during the post-cognitive reappraisal, achieved better accuracy than the pre-cognitive reappraisal case. Considering that the resting data is easier collected than the data under tasks, we conclude from the current study that resting state following cognitive reappraisal might provide potential as a clinical biomarker as well as a reliable tool for the study of structural-functional mechanisms underlying large-scale brain network deterioration in mild cognitive impairment.

## Data Availability Statement

The raw data supporting the conclusions of this article will be made available by the authors, without undue reservation.

## Ethics Statement

The studies involving human participants were reviewed and approved by the Ethics Committee for Clinical Research in Tongji Hospital. The patients/participants provided their written informed consent to participate in this study.

## Author Contributions

YJL, YXL, and NY conceived and planned the study. YXL, ML, RL, WZ, and XZ helped with the subject recruitment and collected the neurophysiological data. SX coded the experiment, together with ML, HL, RL, XC, WZ, and XZ collected the EEG data, and performed the data preprocessing. NY performed the analysis and drafted and wrote the manuscript. YJL helped to write the manuscript. All authors discussed, commented on the results, and contributed to the manuscript.

## Conflict of Interest

The authors declare that the research was conducted in the absence of any commercial or financial relationships that could be construed as a potential conflict of interest.

## Publisher’s Note

All claims expressed in this article are solely those of the authors and do not necessarily represent those of their affiliated organizations, or those of the publisher, the editors and the reviewers. Any product that may be evaluated in this article, or claim that may be made by its manufacturer, is not guaranteed or endorsed by the publisher.
