# Supplementary material for: Functional Brain Networks in Mild Cognitive Impairment Based on Resting Electroencephalography Signals
Source: Front Comput Neurosci. 2021 Oct 20;15:698386. doi: 10.3389/fncom.2021.698386 (PMC8579961; doi:10.3389/fncom.2021.698386)
Supplement: Supplementary file 1 [file Data_Sheet_1.docx]

Supplementary Material

“A Functional brain networks in mild cognitive impairment based on resting EEG signals”

**Nadia Youssef^1^, Shasha Xiao^1^, Meng Liu ^2^, Haipeng Lian^1^, Renren Li ^2^, Xi Chen ^1^,Wei Zhang ^2^, Xiaoran Zheng ^2^,Yunxia Li^2^**, Yingjie Li^1^***

* Correspondence:

Yingjie Li

liyj@i.shu.edu.cn

** Co-Correspondence:

Yunxia Li

doctorLiyunxia@163.com

**Methods**

The MCI participants underwent a standardized diagnostic program including a full physical examination, MRI scan or cranial CT, laboratory screening for treponema pallidum, vitamin B12, free tetraiodothyronine (FT4), thyroid function (free triiodothyronine (FT3), folic acid, thyroid-stimulating hormone (TSH)), and HIV antibodies. The subjects taking medicines (antideperssants, cholinesterase inhibitors and hypnotics), with signs of depression, with dementia, mental illness, history of stroke, or Parkinson’s disease were also excluded.

Memory function was assessed by the Hopkins Verbal Learning Test-Revised (HVLT-R, including immediate recall test, the 5-minutes delayed recall, and the 20-minutes delayed recall test), and the logical memory test (Wechsler memory scale). Language function was measured by the Verbal Fluency Test and the Boston Naming Test (BNT; the 30-item version). Executive function was assessed by the Shape Trail Test-A and B (STT-A, STT-B). Visual space navigation function was measured by the Rey-Osterrieth Complex Figure Test (CFT, including the copy test and the recall test).

How calm or aroused ? 1 2 3 4 5 6 7 8 9

How negative or positive? 1 2 3 4 5 6 7 8 9

**+**

**+**

The soldier is telling the child to leave the battlefield


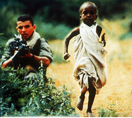


Pre-task resting state

Fixation

Reading time

Arousal rating

Fixation

Post-task resting state

Valence rating

IAPS Image

Time

5 minutes 1 second 8-10 seconds 1 second 5 seconds Unlimited Unlimited 5 minutes


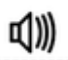


**Figure 1**. Schematic sequence diagram of the experimental paradigm in each trial. Each trial consists of a 5 -minutes resting period, cognitive reappraisal task, and a 5-minutes resting period. A black fixation cross showed up in the center of a gray screen for 1 second, followed by a descriptions of the upcoming picture. The descriptions would be read out (3-5 seconds) and when the sound is over, there were more 5 seconds reading time for subjects to comprehend its meaning. After reading, a black fixation cross was presented on a black screen for 1 seconds telling subjects that reading time was over and attracting their attention to the center of the screen. A picture (either Neutral, Negative, or Reappraisal) was then displayed for 5 seconds. Following each picture, participants were asked to rate each picture on valence (1=negative, 9=positive) and arousal (1=calm, 9=aroused) separately.


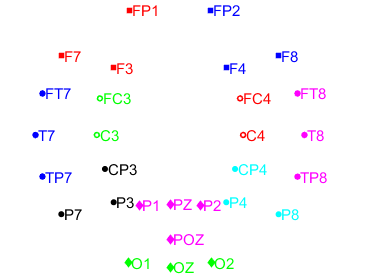

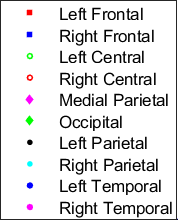


**Figure 2**. Distribution of EEG channels into ten brain regions. the connectivity strength was averaged over the following regions of interests: (1) left prefrontal lobe (LF) (F7, F3, FP1); (2) right prefrontal lobe (RF) (F8, F4, FP2); (3) left temporal lobe (LT) (FT7, T7, TP7); (4) right temporal lobe (RT) (FT8, T8, TP8); (5) left parietal lobe (LP) (CP3, P7, P3); (6) right parietal lobe (RP) (CP4, P4, P8) ;(7) left center (LC) (FC3, C3); (8) right center (RC) (FC4, C4); (9) medial parietal (MP) (P1,P2, Pz, POz); (10) occipital (O) (O1,O2,Oz).

**Table 1. Minimum spanning tree measures summary.**

| *MST graph parameter* | *Abbreviation* | *Description* |
| --- | --- | --- |
| Leaf number | Lf | Number of end nodes (i.e., nodes with degree k=1) |
| Eccentricity | Ecc | Largest distance between a reference node and any other node |
| Diameter | D | Largest path length in a minimum spanning tree |
| Betweenness centrality | BC | Fraction of all shortest paths of shortest paths passing through a particular node |
| Maximum degree | K_max_ | Largest degree in the graph |
| Tree hierarchy | T_h_ | Trade-off between the large scale integration in the MST and the overload of central nodes |

**Table 2. DWPLI connectivity values between different brain regions in pre-task resting state.**

| Frequency band | Interregional connections | dWPLI | | | | p-value | Corrected p-value |
| --- | --- | --- | --- | --- | --- | --- | --- |
|  |  | aMCI |  | Control |  |  |  |
|  |  | Mean | SD | Mean | SD |  |  |
| delta | LF RF | 0.0488 | 0.0296 | 0.06 | 0.0507 | 0.3911 | 0.93 |
|  | LF LC | 0.0556 | 0.0432 | 0.0617 | 0.0502 | 0.6164 | 0.93 |
|  | LF RC | 0.0406 | 0.0353 | 0.0477 | 0.0424 | 0.4756 | 0.93 |
|  | LF MP | 0.0482 | 0.0375 | 0.0515 | 0.0484 | 0.7905 | 0.93 |
|  | LF O | 0.0468 | 0.0432 | 0.0581 | 0.0515 | 0.2217 | 0.93 |
|  | LF LT | 0.054 | 0.0373 | 0.0519 | 0.045 | 0.3238 | 0.93 |
|  | LF RT | 0.0483 | 0.0406 | 0.0505 | 0.0367 | 0.4389 | 0.93 |
|  | LF LP | 0.048 | 0.0359 | 0.0503 | 0.0474 | 0.9094 | 0.93 |
|  | LF RP | 0.0475 | 0.039 | 0.0558 | 0.0625 | 0.7384 | 0.93 |
|  | RF LC | 0.0581 | 0.0456 | 0.0601 | 0.046 | 0.9094 | 0.93 |
|  | RF RC | 0.0413 | 0.0377 | 0.0483 | 0.0544 | 0.5337 | 0.93 |
|  | RF MP | 0.0481 | 0.0382 | 0.0583 | 0.0538 | 0.4524 | 0.93 |
|  | RF O | 0.0503 | 0.0408 | 0.0571 | 0.0449 | 0.3313 | 0.93 |
|  | RF LT | 0.0474 | 0.0388 | 0.0507 | 0.0453 | 0.7499 | 0.93 |
|  | RF RT | 0.0455 | 0.0323 | 0.0531 | 0.0463 | 0.773 | 0.93 |
|  | RF LP | 0.0488 | 0.0341 | 0.0538 | 0.0498 | 0.8081 | 0.93 |
|  | LF RP | 0.0536 | 0.0409 | 0.0656 | 0.0705 | 0.71 | 0.93 |
|  | LC RC | 0.0498 | 0.0489 | 0.0495 | 0.0576 | 0.4081 | 0.93 |
|  | LC MP | 0.0476 | 0.0338 | 0.0589 | 0.0633 | 0.5952 | 0.93 |
|  | LC O | 0.0492 | 0.0452 | 0.0657 | 0.0608 | 0.3585 | 0.93 |
|  | LC LT | 0.0534 | 0.0372 | 0.057 | 0.0602 | 0.4124 | 0.93 |
|  | LC RT | 0.052 | 0.0377 | 0.0628 | 0.0744 | 0.9637 | 0.9637 |
|  | LC LP | 0.0471 | 0.0412 | 0.0521 | 0.0369 | 0.3276 | 0.93 |
|  | LC RP | 0.0514 | 0.0442 | 0.0701 | 0.0709 | 0.143 | 0.93 |
|  | RC MP | 0.0475 | 0.037 | 0.0558 | 0.0693 | 0.8199 | 0.93 |
|  | RC O | 0.0436 | 0.0418 | 0.0498 | 0.047 | 0.4946 | 0.93 |
|  | RC LT | 0.0414 | 0.0284 | 0.0564 | 0.0617 | 0.6326 | 0.93 |
|  | RC RT | 0.0408 | 0.0288 | 0.0529 | 0.0593 | 0.8794 | 0.93 |
|  | RC LP | 0.0384 | 0.0229 | 0.0454 | 0.044 | 0.8081 | 0.93 |
|  | RC RP | 0.0448 | 0.0369 | 0.0617 | 0.0919 | 0.8853 | 0.93 |
|  | MP O | 0.0495 | 0.0396 | 0.0568 | 0.0422 | 0.2581 | 0.93 |
|  | MP LT | 0.0499 | 0.0428 | 0.0608 | 0.0638 | 0.5539 | 0.93 |
|  | MP RT | 0.0442 | 0.0289 | 0.054 | 0.053 | 0.8853 | 0.93 |
|  | MP LP | 0.0447 | 0.0344 | 0.0487 | 0.0428 | 0.7557 | 0.93 |
|  | MP RP | 0.0502 | 0.0395 | 0.0594 | 0.0615 | 0.5189 | 0.93 |
|  | O LT | 0.0465 | 0.0336 | 0.054 | 0.0465 | 0.5238 | 0.93 |
|  | O RT | 0.0454 | 0.0448 | 0.0512 | 0.048 | 0.559 | 0.93 |
|  | O LP | 0.0486 | 0.0358 | 0.0469 | 0.03 | 0.8614 | 0.93 |
|  | O RP | 0.0492 | 0.0413 | 0.059 | 0.0603 | 0.3911 | 0.93 |
|  | LT RT | 0.0498 | 0.0337 | 0.0667 | 0.0719 | 0.5287 | 0.93 |
|  | LT LP | 0.0448 | 0.0296 | 0.0548 | 0.0469 | 0.5952 | 0.93 |
|  | LT RP | 0.0489 | 0.0372 | 0.0668 | 0.0768 | 0.5488 | 0.93 |
|  | RT LP | 0.0471 | 0.0307 | 0.0526 | 0.05 | 0.7043 | 0.93 |
|  | RT RP | 0.0489 | 0.0403 | 0.0679 | 0.0867 | 0.457 | 0.93 |
|  | LP RP | 0.0413 | 0.0278 | 0.0598 | 0.0555 | 0.0809 | 0.93 |
| theta | LF RF | 0.1193 | 0.0766 | 0.1456 | 0.1 | 0.2305 | 0.253 |
|  | LF LC | 0.1333 | 0.1038 | 0.162 | 0.1082 | 0.1162 | 0.1538 |
|  | LF RC | 0.0922 | 0.068 | 0.1322 | 0.0968 | 0.0435 | 0.0783 |
|  | LF MP | 0.1087 | 0.0644 | 0.1261 | 0.0783 | 0.3164 | 0.3236 |
|  | LF O | 0.0949 | 0.0695 | 0.1364 | 0.0842 | 0.0027 | 0.0408 |
|  | LF LT | 0.1093 | 0.0631 | 0.1332 | 0.083 | 0.1918 | 0.2157 |
|  | LF RT | 0.1063 | 0.0739 | 0.1557 | 0.101 | 0.0101 | 0.0527 |
|  | LF LP | 0.1144 | 0.0773 | 0.1532 | 0.0883 | 0.019 | 0.0548 |
|  | LF RP | 0.1238 | 0.0809 | 0.1467 | 0.0871 | 0.1918 | 0.2157 |
|  | RF LC | 0.134 | 0.0862 | 0.1741 | 0.1193 | 0.1216 | 0.1563 |
|  | RF RC | 0.1129 | 0.0805 | 0.1491 | 0.099 | 0.0877 | 0.1316 |
|  | RF MP | 0.1288 | 0.0955 | 0.1466 | 0.0792 | 0.1349 | 0.1686 |
|  | RF O | 0.0951 | 0.0687 | 0.1345 | 0.091 | 0.0288 | 0.0613 |
|  | RF LT | 0.1124 | 0.0694 | 0.1293 | 0.0784 | 0.3313 | 0.3313 |
|  | RF RT | 0.1192 | 0.0762 | 0.1548 | 0.0997 | 0.063 | 0.0977 |
|  | RF LP | 0.1187 | 0.0773 | 0.1489 | 0.1 | 0.1144 | 0.1538 |
|  | LF RP | 0.1463 | 0.1074 | 0.1646 | 0.0954 | 0.2581 | 0.2701 |
|  | LC RC | 0.0927 | 0.0676 | 0.1476 | 0.1056 | 0.0097 | 0.0527 |
|  | LC MP | 0.1059 | 0.0673 | 0.1524 | 0.0814 | 0.0027 | 0.0408 |
|  | LC O | 0.0911 | 0.0769 | 0.1412 | 0.1009 | 0.0056 | 0.0527 |
|  | LC LT | 0.1373 | 0.1002 | 0.1676 | 0.0931 | 0.0619 | 0.0977 |
|  | LC RT | 0.1299 | 0.0916 | 0.1733 | 0.0955 | 0.0175 | 0.0548 |
|  | LC LP | 0.1196 | 0.0918 | 0.1591 | 0.0961 | 0.0152 | 0.0548 |
|  | LC RP | 0.1209 | 0.0843 | 0.1706 | 0.0876 | 0.002 | 0.0408 |
|  | RC MP | 0.0957 | 0.0709 | 0.1233 | 0.0846 | 0.042 | 0.0783 |
|  | RC O | 0.0909 | 0.0603 | 0.1318 | 0.0977 | 0.0412 | 0.0783 |
|  | RC LT | 0.0944 | 0.0637 | 0.1193 | 0.0782 | 0.1127 | 0.1538 |
|  | RC RT | 0.0917 | 0.0677 | 0.1321 | 0.0885 | 0.0128 | 0.0548 |
|  | RC LP | 0.0984 | 0.0726 | 0.1359 | 0.0907 | 0.03 | 0.0613 |
|  | RC RP | 0.102 | 0.0745 | 0.1409 | 0.1019 | 0.052 | 0.09 |
|  | MP O | 0.109 | 0.0689 | 0.1272 | 0.0691 | 0.1451 | 0.1764 |
|  | MP LT | 0.1077 | 0.0722 | 0.1358 | 0.0701 | 0.0161 | 0.0548 |
|  | MP RT | 0.1191 | 0.0862 | 0.1464 | 0.0744 | 0.0215 | 0.0548 |
|  | MP LP | 0.1219 | 0.0864 | 0.1456 | 0.0829 | 0.111 | 0.1538 |
|  | MP RP | 0.1238 | 0.0937 | 0.1519 | 0.0772 | 0.0219 | 0.0548 |
|  | O LT | 0.0968 | 0.0519 | 0.1328 | 0.075 | 0.0257 | 0.0608 |
|  | O RT | 0.0982 | 0.0605 | 0.1369 | 0.0853 | 0.0215 | 0.0548 |
|  | O LP | 0.1035 | 0.0623 | 0.1237 | 0.0756 | 0.2395 | 0.2566 |
|  | O RP | 0.1107 | 0.0816 | 0.1377 | 0.0846 | 0.0619 | 0.0977 |
|  | LT RT | 0.1073 | 0.0755 | 0.1458 | 0.0792 | 0.0076 | 0.0527 |
|  | LT LP | 0.0976 | 0.0665 | 0.1435 | 0.0908 | 0.0105 | 0.0527 |
|  | LT RP | 0.116 | 0.0723 | 0.1535 | 0.0824 | 0.0172 | 0.0548 |
|  | RT LP | 0.1099 | 0.0721 | 0.1503 | 0.081 | 0.009 | 0.0527 |
|  | RT RP | 0.123 | 0.0909 | 0.1642 | 0.0979 | 0.0272 | 0.0612 |
|  | LP RP | 0.1391 | 0.0987 | 0.1654 | 0.1007 | 0.1743 | 0.2064 |
| low alpha | LF RF | 0.3177 | 0.2096 | 0.3594 | 0.227 | 0.5042 | 0.5534 |
|  | LF LC | 0.2757 | 0.2261 | 0.3297 | 0.2296 | 0.1996 | 0.3098 |
|  | LF RC | 0.2325 | 0.173 | 0.276 | 0.216 | 0.4616 | 0.535 |
|  | LF MP | 0.2822 | 0.1716 | 0.2919 | 0.1624 | 0.6598 | 0.6905 |
|  | LF O | 0.2023 | 0.1404 | 0.2709 | 0.1906 | 0.095 | 0.2967 |
|  | LF LT | 0.277 | 0.206 | 0.3084 | 0.2105 | 0.4663 | 0.535 |
|  | LF RT | 0.2813 | 0.2012 | 0.3459 | 0.2255 | 0.1743 | 0.3069 |
|  | LF LP | 0.2994 | 0.1994 | 0.3556 | 0.2137 | 0.197 | 0.3098 |
|  | LF RP | 0.3077 | 0.2099 | 0.3407 | 0.1956 | 0.3506 | 0.4507 |
|  | RF LC | 0.3263 | 0.2181 | 0.3965 | 0.2421 | 0.1816 | 0.3069 |
|  | RF RC | 0.3184 | 0.2063 | 0.3636 | 0.2497 | 0.4756 | 0.535 |
|  | RF MP | 0.3572 | 0.2312 | 0.3724 | 0.2168 | 0.6218 | 0.6662 |
|  | RF O | 0.2232 | 0.1382 | 0.3045 | 0.2138 | 0.1369 | 0.2967 |
|  | RF LT | 0.2978 | 0.2057 | 0.3526 | 0.2313 | 0.2456 | 0.3684 |
|  | RF RT | 0.3232 | 0.1966 | 0.3996 | 0.2464 | 0.1816 | 0.3069 |
|  | RF LP | 0.3023 | 0.2031 | 0.3786 | 0.2384 | 0.1369 | 0.2967 |
|  | LF RP | 0.3545 | 0.2554 | 0.4043 | 0.2546 | 0.3389 | 0.4486 |
|  | LC RC | 0.2039 | 0.1726 | 0.328 | 0.2632 | 0.0283 | 0.2851 |
|  | LC MP | 0.2675 | 0.171 | 0.3174 | 0.2053 | 0.2711 | 0.3859 |
|  | LC O | 0.2099 | 0.1529 | 0.2778 | 0.2051 | 0.1558 | 0.3049 |
|  | LC LT | 0.297 | 0.2512 | 0.3856 | 0.2462 | 0.0459 | 0.2851 |
|  | LC RT | 0.3319 | 0.2464 | 0.3953 | 0.2559 | 0.1841 | 0.3069 |
|  | LC LP | 0.2902 | 0.2118 | 0.3796 | 0.2216 | 0.033 | 0.2851 |
|  | LC RP | 0.2624 | 0.1861 | 0.3222 | 0.1922 | 0.1216 | 0.2967 |
|  | RC MP | 0.2494 | 0.1615 | 0.2461 | 0.1412 | 0.9697 | 0.9697 |
|  | RC O | 0.1871 | 0.1306 | 0.2653 | 0.2064 | 0.1234 | 0.2967 |
|  | RC LT | 0.2459 | 0.1888 | 0.2899 | 0.2148 | 0.3746 | 0.4682 |
|  | RC RT | 0.2264 | 0.1798 | 0.316 | 0.2134 | 0.0323 | 0.2851 |
|  | RC LP | 0.2545 | 0.1727 | 0.327 | 0.2218 | 0.1451 | 0.2967 |
|  | RC RP | 0.2752 | 0.188 | 0.2927 | 0.1949 | 0.6764 | 0.6917 |
|  | MP O | 0.2205 | 0.1234 | 0.2584 | 0.1687 | 0.4616 | 0.535 |
|  | MP LT | 0.2896 | 0.174 | 0.3335 | 0.1911 | 0.2744 | 0.3859 |
|  | MP RT | 0.2711 | 0.1733 | 0.334 | 0.1846 | 0.0663 | 0.2851 |
|  | MP LP | 0.2712 | 0.1815 | 0.3303 | 0.1823 | 0.0697 | 0.2851 |
|  | MP RP | 0.2775 | 0.1864 | 0.334 | 0.1912 | 0.1216 | 0.2967 |
|  | O LT | 0.2218 | 0.128 | 0.2982 | 0.2079 | 0.1409 | 0.2967 |
|  | O RT | 0.2047 | 0.1239 | 0.3055 | 0.2187 | 0.0685 | 0.2851 |
|  | O LP | 0.2062 | 0.1182 | 0.284 | 0.1857 | 0.0663 | 0.2851 |
|  | O RP | 0.2111 | 0.1328 | 0.2607 | 0.189 | 0.3276 | 0.4467 |
|  | LT RT | 0.2772 | 0.1989 | 0.3408 | 0.2058 | 0.1011 | 0.2967 |
|  | LT LP | 0.2676 | 0.1868 | 0.3544 | 0.2294 | 0.0608 | 0.2851 |
|  | LT RP | 0.2794 | 0.1724 | 0.3539 | 0.2078 | 0.1127 | 0.2967 |
|  | RT LP | 0.2377 | 0.1585 | 0.3505 | 0.1968 | 0.0074 | 0.2851 |
|  | RT RP | 0.2926 | 0.199 | 0.3753 | 0.2222 | 0.0697 | 0.2851 |
|  | LP RP | 0.3162 | 0.2319 | 0.3742 | 0.2215 | 0.1216 | 0.2967 |
| upper alpha | LF RF | 0.2773 | 0.1626 | 0.3114 | 0.1844 | 0.3828 | 0.7289 |
|  | LF LC | 0.2452 | 0.1726 | 0.301 | 0.2191 | 0.3313 | 0.7289 |
|  | LF RC | 0.2006 | 0.1439 | 0.2249 | 0.1624 | 0.5238 | 0.7518 |
|  | LF MP | 0.2484 | 0.1476 | 0.2438 | 0.1565 | 0.6875 | 0.8115 |
|  | LF O | 0.1818 | 0.1265 | 0.2216 | 0.1625 | 0.2846 | 0.7289 |
|  | LF LT | 0.2281 | 0.1671 | 0.241 | 0.1714 | 0.7557 | 0.8294 |
|  | LF RT | 0.2355 | 0.1755 | 0.265 | 0.1836 | 0.4168 | 0.7289 |
|  | LF LP | 0.2497 | 0.1437 | 0.2995 | 0.1701 | 0.1493 | 0.7289 |
|  | LF RP | 0.2804 | 0.1708 | 0.2839 | 0.1879 | 0.9335 | 0.9547 |
|  | RF LC | 0.3034 | 0.1824 | 0.3425 | 0.214 | 0.4898 | 0.7518 |
|  | RF RC | 0.2691 | 0.1759 | 0.3031 | 0.1878 | 0.4479 | 0.7419 |
|  | RF MP | 0.3098 | 0.1827 | 0.3157 | 0.1884 | 0.9576 | 0.9576 |
|  | RF O | 0.2023 | 0.1276 | 0.2458 | 0.1804 | 0.3665 | 0.7289 |
|  | RF LT | 0.2616 | 0.1687 | 0.2973 | 0.2034 | 0.5848 | 0.7518 |
|  | RF RT | 0.2735 | 0.1525 | 0.3492 | 0.2092 | 0.1043 | 0.6736 |
|  | RF LP | 0.2708 | 0.1546 | 0.3277 | 0.2053 | 0.2549 | 0.7289 |
|  | LF RP | 0.3109 | 0.1991 | 0.3386 | 0.2128 | 0.7043 | 0.8115 |
|  | LC RC | 0.1734 | 0.1527 | 0.2508 | 0.2001 | 0.0721 | 0.6486 |
|  | LC MP | 0.2392 | 0.1489 | 0.264 | 0.1799 | 0.7213 | 0.8115 |
|  | LC O | 0.1857 | 0.1371 | 0.2253 | 0.1628 | 0.2812 | 0.7289 |
|  | LC LT | 0.2872 | 0.2245 | 0.3123 | 0.222 | 0.4211 | 0.7289 |
|  | LC RT | 0.3023 | 0.2264 | 0.3235 | 0.2162 | 0.5387 | 0.7518 |
|  | LC LP | 0.2642 | 0.1898 | 0.306 | 0.1873 | 0.2023 | 0.7289 |
|  | LC RP | 0.2432 | 0.1497 | 0.2705 | 0.1757 | 0.5744 | 0.7518 |
|  | RC MP | 0.211 | 0.1337 | 0.2199 | 0.1376 | 0.7789 | 0.8345 |
|  | RC O | 0.1602 | 0.1247 | 0.1988 | 0.1487 | 0.288 | 0.7289 |
|  | RC LT | 0.1994 | 0.1679 | 0.2313 | 0.179 | 0.3585 | 0.7289 |
|  | RC RT | 0.1851 | 0.148 | 0.2378 | 0.1644 | 0.1197 | 0.6736 |
|  | RC LP | 0.213 | 0.1322 | 0.2542 | 0.174 | 0.3953 | 0.7289 |
|  | RC RP | 0.2339 | 0.1507 | 0.2438 | 0.1661 | 0.9094 | 0.9517 |
|  | MP O | 0.1874 | 0.1162 | 0.2061 | 0.1341 | 0.5744 | 0.7518 |
|  | MP LT | 0.2499 | 0.1531 | 0.2844 | 0.1794 | 0.4168 | 0.7289 |
|  | MP RT | 0.2323 | 0.1578 | 0.2702 | 0.1777 | 0.5387 | 0.7518 |
|  | MP LP | 0.2206 | 0.1513 | 0.2467 | 0.1599 | 0.4616 | 0.7419 |
|  | MP RP | 0.2362 | 0.1305 | 0.2657 | 0.1835 | 0.6764 | 0.8115 |
|  | O LT | 0.1716 | 0.1029 | 0.2312 | 0.1685 | 0.111 | 0.6736 |
|  | O RT | 0.1595 | 0.1231 | 0.228 | 0.1892 | 0.0468 | 0.6486 |
|  | O LP | 0.1667 | 0.1046 | 0.2235 | 0.1798 | 0.2915 | 0.7289 |
|  | O RP | 0.1861 | 0.1146 | 0.2224 | 0.1742 | 0.6931 | 0.8115 |
|  | LT RT | 0.217 | 0.1616 | 0.2779 | 0.1791 | 0.0641 | 0.6486 |
|  | LT LP | 0.2106 | 0.1458 | 0.2924 | 0.1912 | 0.0412 | 0.6486 |
|  | LT RP | 0.2452 | 0.1616 | 0.3059 | 0.2013 | 0.1719 | 0.7289 |
|  | RT LP | 0.1979 | 0.1219 | 0.2976 | 0.1908 | 0.019 | 0.6486 |
|  | RT RP | 0.2529 | 0.1717 | 0.3099 | 0.2233 | 0.4211 | 0.7289 |
|  | LP RP | 0.2624 | 0.1874 | 0.2989 | 0.2016 | 0.3665 | 0.7289 |
| beta | LF RF | 0.0992 | 0.0554 | 0.1035 | 0.0558 | 0.7156 | 0.979 |
|  | LF LC | 0.1043 | 0.0707 | 0.104 | 0.0602 | 0.8081 | 0.9828 |
|  | LF RC | 0.0701 | 0.0424 | 0.0828 | 0.0557 | 0.3467 | 0.979 |
|  | LF MP | 0.0766 | 0.0461 | 0.0784 | 0.0522 | 0.8436 | 0.999 |
|  | LF O | 0.0944 | 0.0542 | 0.0961 | 0.0635 | 0.9094 | 1 |
|  | LF LT | 0.0814 | 0.0501 | 0.0824 | 0.0469 | 0.7327 | 0.979 |
|  | LF RT | 0.0727 | 0.0403 | 0.081 | 0.0474 | 0.59 | 0.979 |
|  | LF LP | 0.0842 | 0.0423 | 0.0831 | 0.0443 | 0.7213 | 0.979 |
|  | LF RP | 0.0944 | 0.0526 | 0.0906 | 0.061 | 0.4994 | 0.979 |
|  | RF LC | 0.1299 | 0.0726 | 0.1213 | 0.0761 | 0.4803 | 0.979 |
|  | RF RC | 0.0768 | 0.0496 | 0.0938 | 0.0599 | 0.1918 | 0.979 |
|  | RF MP | 0.107 | 0.0598 | 0.1164 | 0.0729 | 0.7327 | 0.979 |
|  | RF O | 0.1067 | 0.0687 | 0.1148 | 0.0679 | 0.559 | 0.979 |
|  | RF LT | 0.1009 | 0.065 | 0.1005 | 0.0686 | 0.7614 | 0.979 |
|  | RF RT | 0.0956 | 0.0577 | 0.1007 | 0.0641 | 0.7384 | 0.979 |
|  | RF LP | 0.1049 | 0.069 | 0.0988 | 0.0542 | 1 | 1 |
|  | LF RP | 0.1195 | 0.0663 | 0.1172 | 0.0727 | 0.6434 | 0.979 |
|  | LC RC | 0.0688 | 0.0465 | 0.0873 | 0.0716 | 0.2023 | 0.979 |
|  | LC MP | 0.0738 | 0.0486 | 0.084 | 0.0592 | 0.5337 | 0.979 |
|  | LC O | 0.0702 | 0.048 | 0.0832 | 0.0545 | 0.2778 | 0.979 |
|  | LC LT | 0.1123 | 0.0853 | 0.1038 | 0.0698 | 0.9879 | 1 |
|  | LC RT | 0.0999 | 0.0634 | 0.0997 | 0.0643 | 0.9576 | 1 |
|  | LC LP | 0.1012 | 0.0621 | 0.09 | 0.0579 | 0.288 | 0.979 |
|  | LC RP | 0.0841 | 0.0477 | 0.0992 | 0.0681 | 0.5796 | 0.979 |
|  | RC MP | 0.0585 | 0.0452 | 0.0686 | 0.0526 | 0.6764 | 0.979 |
|  | RC O | 0.0641 | 0.0456 | 0.0768 | 0.0539 | 0.288 | 0.979 |
|  | RC LT | 0.0661 | 0.0432 | 0.0823 | 0.0539 | 0.1197 | 0.979 |
|  | RC RT | 0.0601 | 0.0359 | 0.0769 | 0.0504 | 0.111 | 0.979 |
|  | RC LP | 0.0635 | 0.0453 | 0.0731 | 0.0482 | 0.2581 | 0.979 |
|  | RC RP | 0.0728 | 0.0541 | 0.0777 | 0.0651 | 0.7964 | 0.9828 |
|  | MP O | 0.0741 | 0.0508 | 0.087 | 0.0576 | 0.3238 | 0.979 |
|  | MP LT | 0.0913 | 0.0696 | 0.0961 | 0.0744 | 0.9818 | 1 |
|  | MP RT | 0.0821 | 0.0568 | 0.0943 | 0.0665 | 0.4255 | 0.979 |
|  | MP LP | 0.0835 | 0.0636 | 0.0924 | 0.0644 | 0.3787 | 0.979 |
|  | MP RP | 0.0997 | 0.0601 | 0.1048 | 0.0722 | 0.9516 | 1 |
|  | O LT | 0.0814 | 0.0621 | 0.0878 | 0.0603 | 0.5952 | 0.979 |
|  | O RT | 0.0719 | 0.0483 | 0.0841 | 0.0588 | 0.4389 | 0.979 |
|  | O LP | 0.0817 | 0.0639 | 0.091 | 0.0594 | 0.2646 | 0.979 |
|  | O RP | 0.0854 | 0.0564 | 0.0844 | 0.0473 | 0.8913 | 1 |
|  | LT RT | 0.0818 | 0.0467 | 0.0901 | 0.0612 | 0.5437 | 0.979 |
|  | LT LP | 0.0818 | 0.0557 | 0.0888 | 0.0591 | 0.6434 | 0.979 |
|  | LT RP | 0.1027 | 0.0738 | 0.1041 | 0.0793 | 0.7442 | 0.979 |
|  | RT LP | 0.0749 | 0.0444 | 0.0853 | 0.0507 | 0.3995 | 0.979 |
|  | RT RP | 0.0883 | 0.0554 | 0.1009 | 0.0748 | 0.559 | 0.979 |
|  | LP RP | 0.1184 | 0.0777 | 0.1046 | 0.0743 | 0.3313 | 0.979 |
| gamma | LF RF | 0.1162 | 0.102 | 0.1035 | 0.085 | 0.4389 | 0.9033 |
|  | LF LC | 0.1124 | 0.0908 | 0.123 | 0.0987 | 0.6931 | 0.9033 |
|  | LF RC | 0.1147 | 0.1026 | 0.1227 | 0.1102 | 0.8794 | 0.9033 |
|  | LF MP | 0.0925 | 0.0908 | 0.0818 | 0.0641 | 0.8376 | 0.9033 |
|  | LF O | 0.1321 | 0.0937 | 0.1418 | 0.0932 | 0.5337 | 0.9033 |
|  | LF LT | 0.1154 | 0.115 | 0.1 | 0.0802 | 0.71 | 0.9033 |
|  | LF RT | 0.1036 | 0.0863 | 0.1087 | 0.0864 | 0.5189 | 0.9033 |
|  | LF LP | 0.1123 | 0.1101 | 0.092 | 0.075 | 0.4803 | 0.9033 |
|  | LF RP | 0.119 | 0.1199 | 0.0866 | 0.0665 | 0.4709 | 0.9033 |
|  | RF LC | 0.1143 | 0.1142 | 0.109 | 0.0926 | 0.8376 | 0.9033 |
|  | RF RC | 0.1087 | 0.1105 | 0.0854 | 0.0829 | 0.2217 | 0.9033 |
|  | RF MP | 0.1012 | 0.0914 | 0.0999 | 0.0796 | 0.9033 | 0.9033 |
|  | RF O | 0.1312 | 0.1011 | 0.1351 | 0.101 | 0.8317 | 0.9033 |
|  | RF LT | 0.1068 | 0.104 | 0.0986 | 0.0736 | 0.8081 | 0.9033 |
|  | RF RT | 0.0971 | 0.0906 | 0.0948 | 0.0735 | 0.8199 | 0.9033 |
|  | RF LP | 0.1204 | 0.1174 | 0.0969 | 0.0769 | 0.8081 | 0.9033 |
|  | LF RP | 0.1329 | 0.1409 | 0.1122 | 0.0866 | 0.8022 | 0.9033 |
|  | LC RC | 0.0942 | 0.0883 | 0.1151 | 0.1071 | 0.3276 | 0.9033 |
|  | LC MP | 0.0838 | 0.0801 | 0.0862 | 0.0888 | 0.8973 | 0.9033 |
|  | LC O | 0.097 | 0.0792 | 0.0884 | 0.0749 | 0.7789 | 0.9033 |
|  | LC LT | 0.1124 | 0.0992 | 0.109 | 0.0873 | 0.7499 | 0.9033 |
|  | LC RT | 0.0966 | 0.0769 | 0.1125 | 0.0984 | 0.3092 | 0.9033 |
|  | LC LP | 0.0967 | 0.0969 | 0.1016 | 0.104 | 0.8614 | 0.9033 |
|  | LC RP | 0.1022 | 0.0996 | 0.0983 | 0.1041 | 0.7043 | 0.9033 |
|  | RC MP | 0.0994 | 0.1101 | 0.0834 | 0.0952 | 0.4709 | 0.9033 |
|  | RC O | 0.1216 | 0.0856 | 0.1136 | 0.0902 | 0.7327 | 0.9033 |
|  | RC LT | 0.1029 | 0.1144 | 0.0978 | 0.0718 | 0.5641 | 0.9033 |
|  | RC RT | 0.1038 | 0.1049 | 0.1056 | 0.0828 | 0.5488 | 0.9033 |
|  | RC LP | 0.1104 | 0.1033 | 0.0958 | 0.1017 | 0.2812 | 0.9033 |
|  | RC RP | 0.1052 | 0.1252 | 0.0776 | 0.0741 | 0.3911 | 0.9033 |
|  | MP O | 0.1134 | 0.0977 | 0.1205 | 0.0902 | 0.6005 | 0.9033 |
|  | MP LT | 0.0749 | 0.0783 | 0.0937 | 0.0919 | 0.3092 | 0.9033 |
|  | MP RT | 0.0906 | 0.0885 | 0.0938 | 0.0811 | 0.638 | 0.9033 |
|  | MP LP | 0.0934 | 0.0885 | 0.1032 | 0.0843 | 0.3869 | 0.9033 |
|  | MP RP | 0.1432 | 0.1203 | 0.1296 | 0.09 | 0.8973 | 0.9033 |
|  | O LT | 0.0842 | 0.0828 | 0.0885 | 0.0638 | 0.1603 | 0.9033 |
|  | O RT | 0.0851 | 0.0809 | 0.0877 | 0.0724 | 0.5238 | 0.9033 |
|  | O LP | 0.1082 | 0.1041 | 0.1034 | 0.076 | 0.71 | 0.9033 |
|  | O RP | 0.1148 | 0.1121 | 0.1143 | 0.0753 | 0.3585 | 0.9033 |
|  | LT RT | 0.095 | 0.1109 | 0.1019 | 0.0651 | 0.0809 | 0.9033 |
|  | LT LP | 0.0939 | 0.1006 | 0.0993 | 0.0805 | 0.2217 | 0.9033 |
|  | LT RP | 0.0996 | 0.1079 | 0.0968 | 0.0786 | 0.6819 | 0.9033 |
|  | RT LP | 0.1064 | 0.1071 | 0.0908 | 0.0688 | 0.7847 | 0.9033 |
|  | RT RP | 0.1064 | 0.1145 | 0.0898 | 0.063 | 0.727 | 0.9033 |
|  | LP RP | 0.1357 | 0.1287 | 0.1169 | 0.0857 | 0.7672 | 0.9033 |

**Table 3. DWPLI connectivity values between different brain regions in post-task resting state.**

| Frequency band | Interregional connections | dWPLI |  |  |  | p-value | Corrected p-value |
| --- | --- | --- | --- | --- | --- | --- | --- |
|  |  | aMCI |  | Control |  |  |  |
|  |  | Mean | SD | Mean | SD |  |  |
| delta | LF RF | 0.0598 | 0.0444 | 0.0571 | 0.0332 | 0.6987 | 0.9832 |
|  | LF LC | 0.0738 | 0.0691 | 0.0663 | 0.0537 | 0.9274 | 0.9832 |
|  | LF RC | 0.0595 | 0.044 | 0.0477 | 0.0367 | 0.2023 | 0.9832 |
|  | LF MP | 0.06 | 0.0458 | 0.0464 | 0.0325 | 0.1216 | 0.9832 |
|  | LF O | 0.0652 | 0.0472 | 0.0584 | 0.0393 | 0.5287 | 0.9832 |
|  | LF LT | 0.0634 | 0.0438 | 0.0582 | 0.0422 | 0.59 | 0.9832 |
|  | LF RT | 0.0597 | 0.0451 | 0.0482 | 0.0337 | 0.1493 | 0.9832 |
|  | LF LP | 0.0582 | 0.039 | 0.0494 | 0.0294 | 0.3092 | 0.9832 |
|  | LF RP | 0.069 | 0.0529 | 0.0481 | 0.03 | 0.0215 | 0.9662 |
|  | RF LC | 0.069 | 0.059 | 0.062 | 0.0466 | 0.7499 | 0.9832 |
|  | RF RC | 0.0517 | 0.044 | 0.0418 | 0.0301 | 0.4344 | 0.9832 |
|  | RF MP | 0.0506 | 0.0364 | 0.0514 | 0.0375 | 0.8614 | 0.9832 |
|  | RF O | 0.0629 | 0.0501 | 0.0643 | 0.0523 | 0.9939 | 0.9939 |
|  | RF LT | 0.0602 | 0.0422 | 0.0576 | 0.037 | 0.9033 | 0.9832 |
|  | RF RT | 0.0587 | 0.039 | 0.0531 | 0.036 | 0.5238 | 0.9832 |
|  | RF LP | 0.0582 | 0.0356 | 0.0493 | 0.0265 | 0.3506 | 0.9832 |
|  | LF RP | 0.0682 | 0.0499 | 0.0555 | 0.0363 | 0.3056 | 0.9832 |
|  | LC RC | 0.0549 | 0.0561 | 0.054 | 0.05 | 0.4756 | 0.9832 |
|  | LC MP | 0.0628 | 0.0527 | 0.0527 | 0.0405 | 0.3506 | 0.9832 |
|  | LC O | 0.0683 | 0.0766 | 0.0648 | 0.0479 | 0.3164 | 0.9832 |
|  | LC LT | 0.0582 | 0.0542 | 0.0608 | 0.0483 | 0.3128 | 0.9832 |
|  | LC RT | 0.0602 | 0.0523 | 0.06 | 0.0437 | 0.638 | 0.9832 |
|  | LC LP | 0.0547 | 0.0471 | 0.0498 | 0.0335 | 0.9637 | 0.9856 |
|  | LC RP | 0.0717 | 0.0608 | 0.0538 | 0.0406 | 0.2518 | 0.9832 |
|  | RC MP | 0.0554 | 0.0682 | 0.0446 | 0.0373 | 0.9214 | 0.9832 |
|  | RC O | 0.0547 | 0.0528 | 0.0471 | 0.0254 | 0.9335 | 0.9832 |
|  | RC LT | 0.0574 | 0.0584 | 0.0538 | 0.0423 | 0.8258 | 0.9832 |
|  | RC RT | 0.0564 | 0.0608 | 0.0514 | 0.0395 | 0.8258 | 0.9832 |
|  | RC LP | 0.0491 | 0.0494 | 0.0458 | 0.035 | 0.8913 | 0.9832 |
|  | RC RP | 0.058 | 0.0584 | 0.0456 | 0.0355 | 0.7442 | 0.9832 |
|  | MP O | 0.0494 | 0.0396 | 0.0555 | 0.0401 | 0.3092 | 0.9832 |
|  | MP LT | 0.0586 | 0.0444 | 0.0523 | 0.0498 | 0.4663 | 0.9832 |
|  | MP RT | 0.0591 | 0.0548 | 0.0549 | 0.0397 | 0.6326 | 0.9832 |
|  | MP LP | 0.0506 | 0.0399 | 0.0489 | 0.0337 | 0.8734 | 0.9832 |
|  | MP RP | 0.06 | 0.0409 | 0.0566 | 0.0411 | 0.6272 | 0.9832 |
|  | O LT | 0.0581 | 0.0437 | 0.062 | 0.042 | 0.4038 | 0.9832 |
|  | O RT | 0.0632 | 0.0549 | 0.0522 | 0.033 | 0.7156 | 0.9832 |
|  | O LP | 0.0549 | 0.0483 | 0.0531 | 0.0309 | 0.2518 | 0.9832 |
|  | O RP | 0.0556 | 0.0385 | 0.0539 | 0.0431 | 0.5238 | 0.9832 |
|  | LT RT | 0.0646 | 0.0532 | 0.0659 | 0.0472 | 0.8853 | 0.9832 |
|  | LT LP | 0.0568 | 0.0406 | 0.0599 | 0.0428 | 0.6987 | 0.9832 |
|  | LT RP | 0.071 | 0.0549 | 0.0586 | 0.0567 | 0.1944 | 0.9832 |
|  | RT LP | 0.0569 | 0.045 | 0.0525 | 0.03 | 0.9395 | 0.9832 |
|  | RT RP | 0.0738 | 0.0571 | 0.0559 | 0.046 | 0.095 | 0.9832 |
|  | LP RP | 0.0578 | 0.035 | 0.0574 | 0.042 | 0.7043 | 0.9832 |
| theta | LF RF | 0.1183 | 0.0763 | 0.1535 | 0.1001 | 0.0849 | 0.1233 |
|  | LF LC | 0.133 | 0.11 | 0.1754 | 0.1265 | 0.0663 | 0.0994 |
|  | LF RC | 0.0966 | 0.0721 | 0.1245 | 0.0948 | 0.1816 | 0.2335 |
|  | LF MP | 0.1069 | 0.0762 | 0.1221 | 0.0864 | 0.4479 | 0.4479 |
|  | LF O | 0.087 | 0.0649 | 0.1291 | 0.0819 | 0.0036 | 0.0321 |
|  | LF LT | 0.1035 | 0.0769 | 0.1311 | 0.0826 | 0.0511 | 0.0852 |
|  | LF RT | 0.1129 | 0.0962 | 0.1537 | 0.0958 | 0.006 | 0.0324 |
|  | LF LP | 0.1066 | 0.0821 | 0.1507 | 0.1045 | 0.021 | 0.0495 |
|  | LF RP | 0.1103 | 0.0881 | 0.1406 | 0.1013 | 0.106 | 0.149 |
|  | RF LC | 0.1366 | 0.1077 | 0.1673 | 0.1153 | 0.1389 | 0.1838 |
|  | RF RC | 0.1027 | 0.0745 | 0.1345 | 0.0965 | 0.0608 | 0.0961 |
|  | RF MP | 0.1324 | 0.0915 | 0.1465 | 0.0786 | 0.2646 | 0.2976 |
|  | RF O | 0.098 | 0.0743 | 0.1366 | 0.0768 | 0.0025 | 0.0321 |
|  | RF LT | 0.1103 | 0.0756 | 0.129 | 0.0836 | 0.2217 | 0.266 |
|  | RF RT | 0.1149 | 0.0898 | 0.155 | 0.0959 | 0.0094 | 0.0324 |
|  | RF LP | 0.1266 | 0.0921 | 0.1405 | 0.0857 | 0.2846 | 0.3049 |
|  | LF RP | 0.1477 | 0.1142 | 0.1553 | 0.0839 | 0.2915 | 0.305 |
|  | LC RC | 0.0982 | 0.0825 | 0.1352 | 0.1159 | 0.1234 | 0.1683 |
|  | LC MP | 0.1171 | 0.088 | 0.1311 | 0.0847 | 0.2846 | 0.3049 |
|  | LC O | 0.1044 | 0.1121 | 0.131 | 0.0929 | 0.0336 | 0.063 |
|  | LC LT | 0.1441 | 0.1165 | 0.1612 | 0.1003 | 0.2246 | 0.266 |
|  | LC RT | 0.1592 | 0.1223 | 0.1809 | 0.1105 | 0.197 | 0.2462 |
|  | LC LP | 0.1198 | 0.1033 | 0.1577 | 0.1059 | 0.0223 | 0.0495 |
|  | LC RP | 0.1445 | 0.1241 | 0.1412 | 0.084 | 0.3787 | 0.3873 |
|  | RC MP | 0.0847 | 0.0686 | 0.11 | 0.0802 | 0.0619 | 0.0961 |
|  | RC O | 0.0897 | 0.0864 | 0.1163 | 0.0788 | 0.0092 | 0.0324 |
|  | RC LT | 0.0996 | 0.0624 | 0.1177 | 0.076 | 0.2549 | 0.2942 |
|  | RC RT | 0.1034 | 0.0837 | 0.1299 | 0.0863 | 0.0412 | 0.0739 |
|  | RC LP | 0.096 | 0.0786 | 0.1294 | 0.0851 | 0.0198 | 0.0495 |
|  | RC RP | 0.0932 | 0.0909 | 0.1275 | 0.0936 | 0.0272 | 0.0532 |
|  | MP O | 0.0963 | 0.0609 | 0.129 | 0.0707 | 0.0077 | 0.0324 |
|  | MP LT | 0.1077 | 0.0716 | 0.1313 | 0.0678 | 0.0427 | 0.0739 |
|  | MP RT | 0.1065 | 0.0904 | 0.1473 | 0.0776 | 0.0018 | 0.0321 |
|  | MP LP | 0.1099 | 0.0773 | 0.1503 | 0.0874 | 0.0077 | 0.0324 |
|  | MP RP | 0.1239 | 0.093 | 0.1537 | 0.0825 | 0.0115 | 0.0345 |
|  | O LT | 0.0933 | 0.068 | 0.1281 | 0.0723 | 0.0016 | 0.0321 |
|  | O RT | 0.1039 | 0.0954 | 0.1281 | 0.0756 | 0.0101 | 0.0324 |
|  | O LP | 0.0894 | 0.0522 | 0.1283 | 0.0722 | 0.0067 | 0.0324 |
|  | O RP | 0.0989 | 0.0701 | 0.1353 | 0.0819 | 0.0242 | 0.0495 |
|  | LT RT | 0.1105 | 0.0829 | 0.1526 | 0.0828 | 0.0029 | 0.0321 |
|  | LT LP | 0.1085 | 0.0875 | 0.1431 | 0.0816 | 0.009 | 0.0324 |
|  | LT RP | 0.1183 | 0.0815 | 0.1527 | 0.0869 | 0.0175 | 0.0464 |
|  | RT LP | 0.1165 | 0.0973 | 0.1422 | 0.0731 | 0.0142 | 0.04 |
|  | RT RP | 0.1293 | 0.1178 | 0.1668 | 0.0906 | 0.0081 | 0.0324 |
|  | LP RP | 0.1303 | 0.088 | 0.1668 | 0.0872 | 0.0242 | 0.0495 |
| low alpha | LF RF | 0.3583 | 0.2345 | 0.3813 | 0.2089 | 0.5692 | 0.7497 |
|  | LF LC | 0.3291 | 0.2489 | 0.3754 | 0.2621 | 0.4616 | 0.7163 |
|  | LF RC | 0.2908 | 0.2144 | 0.2977 | 0.2041 | 0.7672 | 0.8334 |
|  | LF MP | 0.3147 | 0.1762 | 0.3095 | 0.1698 | 0.7964 | 0.8334 |
|  | LF O | 0.2275 | 0.1566 | 0.289 | 0.192 | 0.1253 | 0.5351 |
|  | LF LT | 0.2738 | 0.1929 | 0.3187 | 0.2012 | 0.2334 | 0.5351 |
|  | LF RT | 0.3159 | 0.2099 | 0.3626 | 0.2161 | 0.2915 | 0.5351 |
|  | LF LP | 0.333 | 0.2026 | 0.3563 | 0.2163 | 0.559 | 0.7497 |
|  | LF RP | 0.3319 | 0.22 | 0.3462 | 0.1933 | 0.6488 | 0.7684 |
|  | RF LC | 0.3434 | 0.2178 | 0.3913 | 0.2282 | 0.3428 | 0.5713 |
|  | RF RC | 0.3534 | 0.2533 | 0.3684 | 0.2348 | 0.5796 | 0.7497 |
|  | RF MP | 0.3674 | 0.2162 | 0.365 | 0.1904 | 0.8913 | 0.8913 |
|  | RF O | 0.2772 | 0.1881 | 0.3228 | 0.2031 | 0.2678 | 0.5351 |
|  | RF LT | 0.3099 | 0.2112 | 0.3623 | 0.2103 | 0.1558 | 0.5351 |
|  | RF RT | 0.3464 | 0.2215 | 0.4175 | 0.2234 | 0.1271 | 0.5351 |
|  | RF LP | 0.3218 | 0.2173 | 0.3773 | 0.2199 | 0.1892 | 0.5351 |
|  | LF RP | 0.383 | 0.2585 | 0.3933 | 0.2146 | 0.6164 | 0.7497 |
|  | LC RC | 0.2773 | 0.2253 | 0.3342 | 0.2592 | 0.2744 | 0.5351 |
|  | LC MP | 0.2992 | 0.1732 | 0.327 | 0.185 | 0.59 | 0.7497 |
|  | LC O | 0.2552 | 0.1883 | 0.3046 | 0.202 | 0.2487 | 0.5351 |
|  | LC LT | 0.3153 | 0.2437 | 0.3647 | 0.2344 | 0.2549 | 0.5351 |
|  | LC RT | 0.3736 | 0.2448 | 0.4279 | 0.2719 | 0.3092 | 0.5351 |
|  | LC LP | 0.3115 | 0.1798 | 0.3633 | 0.2094 | 0.2549 | 0.5351 |
|  | LC RP | 0.2968 | 0.2065 | 0.3226 | 0.1788 | 0.2711 | 0.5351 |
|  | RC MP | 0.2659 | 0.1932 | 0.2792 | 0.1712 | 0.4994 | 0.7249 |
|  | RC O | 0.2158 | 0.1904 | 0.2613 | 0.1842 | 0.1093 | 0.5351 |
|  | RC LT | 0.251 | 0.1829 | 0.3058 | 0.2094 | 0.2613 | 0.5351 |
|  | RC RT | 0.2615 | 0.1684 | 0.3275 | 0.198 | 0.1349 | 0.5351 |
|  | RC LP | 0.2807 | 0.19 | 0.3286 | 0.2157 | 0.2812 | 0.5351 |
|  | RC RP | 0.2685 | 0.2104 | 0.3034 | 0.201 | 0.2985 | 0.5351 |
|  | MP O | 0.249 | 0.1469 | 0.2686 | 0.1724 | 0.7499 | 0.8334 |
|  | MP LT | 0.3066 | 0.1846 | 0.33 | 0.1989 | 0.7442 | 0.8334 |
|  | MP RT | 0.3004 | 0.1943 | 0.3442 | 0.2031 | 0.3746 | 0.602 |
|  | MP LP | 0.31 | 0.1852 | 0.3121 | 0.1693 | 0.7964 | 0.8334 |
|  | MP RP | 0.3252 | 0.1821 | 0.3288 | 0.1715 | 0.8794 | 0.8913 |
|  | O LT | 0.236 | 0.1799 | 0.2944 | 0.2128 | 0.2077 | 0.5351 |
|  | O RT | 0.2361 | 0.1816 | 0.2898 | 0.204 | 0.1743 | 0.5351 |
|  | O LP | 0.2381 | 0.1733 | 0.2658 | 0.1787 | 0.6058 | 0.7497 |
|  | O RP | 0.2458 | 0.189 | 0.278 | 0.1948 | 0.3056 | 0.5351 |
|  | LT RT | 0.2979 | 0.1917 | 0.3778 | 0.2085 | 0.0619 | 0.5351 |
|  | LT LP | 0.2936 | 0.1828 | 0.3457 | 0.199 | 0.2305 | 0.5351 |
|  | LT RP | 0.3035 | 0.2146 | 0.3519 | 0.2078 | 0.2334 | 0.5351 |
|  | RT LP | 0.3081 | 0.2106 | 0.3506 | 0.1877 | 0.1892 | 0.5351 |
|  | RT RP | 0.3299 | 0.232 | 0.3904 | 0.2229 | 0.1349 | 0.5351 |
|  | LP RP | 0.3477 | 0.2161 | 0.3724 | 0.2177 | 0.4898 | 0.7249 |
| upper alpha | LF RF | 0.3292 | 0.2066 | 0.2986 | 0.1818 | 0.457 | 1 |
|  | LF LC | 0.3052 | 0.2326 | 0.2923 | 0.2134 | 1 | 1 |
|  | LF RC | 0.2483 | 0.1798 | 0.2305 | 0.1693 | 0.773 | 1 |
|  | LF MP | 0.2763 | 0.1626 | 0.241 | 0.1608 | 0.2613 | 1 |
|  | LF O | 0.1981 | 0.1268 | 0.2133 | 0.158 | 0.9516 | 1 |
|  | LF LT | 0.2474 | 0.1774 | 0.2327 | 0.1692 | 0.7043 | 1 |
|  | LF RT | 0.2776 | 0.2016 | 0.2666 | 0.1685 | 0.8614 | 1 |
|  | LF LP | 0.2907 | 0.1625 | 0.2821 | 0.1687 | 0.7156 | 1 |
|  | LF RP | 0.3142 | 0.1939 | 0.2636 | 0.1792 | 0.197 | 1 |
|  | RF LC | 0.3206 | 0.2202 | 0.3154 | 0.2112 | 0.9697 | 1 |
|  | RF RC | 0.3217 | 0.2148 | 0.286 | 0.1793 | 0.5042 | 1 |
|  | RF MP | 0.3138 | 0.1987 | 0.2952 | 0.1861 | 0.6819 | 1 |
|  | RF O | 0.2448 | 0.1543 | 0.2395 | 0.1673 | 0.7213 | 1 |
|  | RF LT | 0.2799 | 0.1908 | 0.2806 | 0.1977 | 0.9576 | 1 |
|  | RF RT | 0.3116 | 0.2069 | 0.3249 | 0.1937 | 0.59 | 1 |
|  | RF LP | 0.2953 | 0.1784 | 0.3065 | 0.1824 | 0.727 | 1 |
|  | LF RP | 0.3273 | 0.2137 | 0.3055 | 0.2007 | 0.5692 | 1 |
|  | LC RC | 0.2415 | 0.2027 | 0.2209 | 0.1914 | 0.6653 | 1 |
|  | LC MP | 0.2591 | 0.1465 | 0.2575 | 0.1663 | 0.6819 | 1 |
|  | LC O | 0.2243 | 0.1463 | 0.2232 | 0.1597 | 0.9033 | 1 |
|  | LC LT | 0.2825 | 0.2274 | 0.2667 | 0.1911 | 0.9576 | 1 |
|  | LC RT | 0.3195 | 0.2373 | 0.2993 | 0.1916 | 0.9637 | 1 |
|  | LC LP | 0.2638 | 0.1823 | 0.277 | 0.1752 | 0.6434 | 1 |
|  | LC RP | 0.251 | 0.1751 | 0.2596 | 0.163 | 0.638 | 1 |
|  | RC MP | 0.2504 | 0.1655 | 0.2187 | 0.1445 | 0.3953 | 1 |
|  | RC O | 0.1836 | 0.1356 | 0.1883 | 0.1503 | 0.8081 | 1 |
|  | RC LT | 0.2163 | 0.166 | 0.2172 | 0.17 | 1 | 1 |
|  | RC RT | 0.2209 | 0.157 | 0.2167 | 0.1562 | 0.9214 | 1 |
|  | RC LP | 0.248 | 0.1525 | 0.2518 | 0.1694 | 0.9094 | 1 |
|  | RC RP | 0.2589 | 0.179 | 0.2247 | 0.1662 | 0.2949 | 1 |
|  | MP O | 0.212 | 0.1158 | 0.1933 | 0.1371 | 0.2549 | 1 |
|  | MP LT | 0.2639 | 0.1812 | 0.2826 | 0.1904 | 0.7964 | 1 |
|  | MP RT | 0.253 | 0.1786 | 0.2729 | 0.181 | 0.6488 | 1 |
|  | MP LP | 0.2472 | 0.1683 | 0.231 | 0.1591 | 0.6434 | 1 |
|  | MP RP | 0.2654 | 0.1518 | 0.265 | 0.1684 | 0.7156 | 1 |
|  | O LT | 0.1956 | 0.1416 | 0.2165 | 0.1701 | 0.6987 | 1 |
|  | O RT | 0.1853 | 0.1532 | 0.2002 | 0.1704 | 0.6875 | 1 |
|  | O LP | 0.1907 | 0.1324 | 0.1944 | 0.1429 | 0.8794 | 1 |
|  | O RP | 0.2155 | 0.1471 | 0.204 | 0.1682 | 0.4081 | 1 |
|  | LT RT | 0.239 | 0.1718 | 0.2769 | 0.1885 | 0.3506 | 1 |
|  | LT LP | 0.233 | 0.1635 | 0.2609 | 0.1756 | 0.5287 | 1 |
|  | LT RP | 0.2584 | 0.1951 | 0.2915 | 0.1966 | 0.3545 | 1 |
|  | RT LP | 0.2393 | 0.1596 | 0.2659 | 0.1706 | 0.4994 | 1 |
|  | RT RP | 0.2887 | 0.2042 | 0.3011 | 0.2068 | 0.8022 | 1 |
|  | LP RP | 0.2947 | 0.204 | 0.2871 | 0.2145 | 0.6326 | 1 |
| beta | LF RF | 0.1132 | 0.0621 | 0.1083 | 0.0682 | 0.5091 | 0.9335 |
|  | LF LC | 0.1095 | 0.071 | 0.1052 | 0.0635 | 0.8614 | 0.9335 |
|  | LF RC | 0.0844 | 0.0623 | 0.0962 | 0.0634 | 0.2915 | 0.9335 |
|  | LF MP | 0.0781 | 0.0539 | 0.0759 | 0.0524 | 0.6708 | 0.9335 |
|  | LF O | 0.0919 | 0.0566 | 0.0988 | 0.0647 | 0.7327 | 0.9335 |
|  | LF LT | 0.1005 | 0.0691 | 0.0889 | 0.0559 | 0.6598 | 0.9335 |
|  | LF RT | 0.0839 | 0.0484 | 0.0853 | 0.0556 | 0.9274 | 0.9335 |
|  | LF LP | 0.0954 | 0.048 | 0.0907 | 0.0637 | 0.3056 | 0.9335 |
|  | LF RP | 0.0981 | 0.0624 | 0.0956 | 0.0668 | 0.6005 | 0.9335 |
|  | RF LC | 0.1354 | 0.0886 | 0.1165 | 0.0766 | 0.3164 | 0.9335 |
|  | RF RC | 0.0908 | 0.0547 | 0.0935 | 0.0637 | 0.8495 | 0.9335 |
|  | RF MP | 0.1148 | 0.0675 | 0.1133 | 0.0699 | 0.6653 | 0.9335 |
|  | RF O | 0.1156 | 0.0659 | 0.1204 | 0.073 | 0.8674 | 0.9335 |
|  | RF LT | 0.1163 | 0.0752 | 0.095 | 0.0642 | 0.106 | 0.9335 |
|  | RF RT | 0.1033 | 0.0624 | 0.1028 | 0.0701 | 0.8555 | 0.9335 |
|  | RF LP | 0.1195 | 0.0637 | 0.1061 | 0.0614 | 0.2217 | 0.9335 |
|  | LF RP | 0.1262 | 0.0694 | 0.1241 | 0.0838 | 0.5387 | 0.9335 |
|  | LC RC | 0.0806 | 0.0636 | 0.0817 | 0.0734 | 0.8614 | 0.9335 |
|  | LC MP | 0.0876 | 0.0762 | 0.0787 | 0.0598 | 0.6058 | 0.9335 |
|  | LC O | 0.0792 | 0.0562 | 0.0799 | 0.0557 | 0.9335 | 0.9335 |
|  | LC LT | 0.136 | 0.1009 | 0.106 | 0.0715 | 0.2275 | 0.9335 |
|  | LC RT | 0.1063 | 0.066 | 0.0979 | 0.0666 | 0.4479 | 0.9335 |
|  | LC LP | 0.1052 | 0.0702 | 0.0821 | 0.0569 | 0.1216 | 0.9335 |
|  | LC RP | 0.0991 | 0.0715 | 0.0979 | 0.0685 | 0.8794 | 0.9335 |
|  | RC MP | 0.0697 | 0.0399 | 0.0686 | 0.0553 | 0.2394 | 0.9335 |
|  | RC O | 0.0703 | 0.0409 | 0.0769 | 0.0574 | 0.9094 | 0.9335 |
|  | RC LT | 0.0844 | 0.0536 | 0.0805 | 0.0572 | 0.6272 | 0.9335 |
|  | RC RT | 0.0708 | 0.0386 | 0.0741 | 0.0527 | 0.7384 | 0.9335 |
|  | RC LP | 0.0734 | 0.039 | 0.0775 | 0.0537 | 0.8436 | 0.9335 |
|  | RC RP | 0.0748 | 0.0481 | 0.0778 | 0.0654 | 0.5189 | 0.9335 |
|  | MP O | 0.0822 | 0.0492 | 0.0837 | 0.0591 | 0.727 | 0.9335 |
|  | MP LT | 0.1014 | 0.0666 | 0.103 | 0.0826 | 0.4479 | 0.9335 |
|  | MP RT | 0.092 | 0.0585 | 0.0909 | 0.0688 | 0.457 | 0.9335 |
|  | MP LP | 0.1041 | 0.0601 | 0.0885 | 0.0579 | 0.1743 | 0.9335 |
|  | MP RP | 0.1161 | 0.0742 | 0.0983 | 0.0739 | 0.1515 | 0.9335 |
|  | O LT | 0.0878 | 0.0617 | 0.0948 | 0.0705 | 0.6764 | 0.9335 |
|  | O RT | 0.0691 | 0.0398 | 0.078 | 0.0587 | 0.6875 | 0.9335 |
|  | O LP | 0.0824 | 0.0469 | 0.0877 | 0.0632 | 0.9094 | 0.9335 |
|  | O RP | 0.09 | 0.0535 | 0.0868 | 0.0678 | 0.3201 | 0.9335 |
|  | LT RT | 0.0955 | 0.0559 | 0.089 | 0.0599 | 0.4663 | 0.9335 |
|  | LT LP | 0.0973 | 0.0569 | 0.0852 | 0.0527 | 0.3128 | 0.9335 |
|  | LT RP | 0.1172 | 0.0712 | 0.1054 | 0.082 | 0.2105 | 0.9335 |
|  | RT LP | 0.0798 | 0.0526 | 0.0808 | 0.0516 | 0.9033 | 0.9335 |
|  | RT RP | 0.1027 | 0.0698 | 0.0952 | 0.0787 | 0.288 | 0.9335 |
|  | LP RP | 0.1306 | 0.0765 | 0.1042 | 0.0756 | 0.0443 | 0.9335 |
| gamma | LF RF | 0.1236 | 0.1044 | 0.1104 | 0.0885 | 0.6708 | 1 |
|  | LF LC | 0.1087 | 0.0958 | 0.1172 | 0.0956 | 0.5488 | 1 |
|  | LF RC | 0.1053 | 0.0802 | 0.1258 | 0.1119 | 0.6005 | 1 |
|  | LF MP | 0.1168 | 0.1122 | 0.0791 | 0.052 | 0.3584 | 1 |
|  | LF O | 0.1403 | 0.099 | 0.1214 | 0.0966 | 0.2581 | 1 |
|  | LF LT | 0.1267 | 0.1002 | 0.1178 | 0.0767 | 0.9939 | 1 |
|  | LF RT | 0.1081 | 0.0756 | 0.1095 | 0.0834 | 0.9637 | 1 |
|  | LF LP | 0.1059 | 0.086 | 0.0994 | 0.0765 | 0.7327 | 1 |
|  | LF RP | 0.1192 | 0.1168 | 0.0915 | 0.0689 | 0.6653 | 1 |
|  | RF LC | 0.124 | 0.1173 | 0.1101 | 0.0796 | 1 | 1 |
|  | RF RC | 0.1058 | 0.1026 | 0.1074 | 0.0902 | 0.5796 | 1 |
|  | RF MP | 0.145 | 0.1513 | 0.0971 | 0.069 | 0.2133 | 1 |
|  | RF O | 0.1463 | 0.1131 | 0.1221 | 0.1049 | 0.197 | 1 |
|  | RF LT | 0.1255 | 0.1002 | 0.1053 | 0.0759 | 0.4434 | 1 |
|  | RF RT | 0.1212 | 0.1022 | 0.1085 | 0.0809 | 0.8436 | 1 |
|  | RF LP | 0.1446 | 0.1206 | 0.1142 | 0.0846 | 0.2646 | 1 |
|  | LF RP | 0.1578 | 0.1517 | 0.1235 | 0.0856 | 0.5387 | 1 |
|  | LC RC | 0.0949 | 0.0931 | 0.1186 | 0.1191 | 0.514 | 1 |
|  | LC MP | 0.1069 | 0.1213 | 0.0756 | 0.0535 | 0.8081 | 1 |
|  | LC O | 0.093 | 0.0771 | 0.0765 | 0.0636 | 0.2518 | 1 |
|  | LC LT | 0.1329 | 0.1119 | 0.1224 | 0.0884 | 0.8853 | 1 |
|  | LC RT | 0.1154 | 0.0876 | 0.1162 | 0.0902 | 0.8734 | 1 |
|  | LC LP | 0.1042 | 0.0947 | 0.1078 | 0.0874 | 0.8081 | 1 |
|  | LC RP | 0.1164 | 0.1279 | 0.094 | 0.0831 | 0.6819 | 1 |
|  | RC MP | 0.0947 | 0.0744 | 0.0728 | 0.0523 | 0.3164 | 1 |
|  | RC O | 0.1138 | 0.0788 | 0.0979 | 0.0706 | 0.3995 | 1 |
|  | RC LT | 0.1102 | 0.0829 | 0.1098 | 0.0808 | 0.9516 | 1 |
|  | RC RT | 0.1155 | 0.0819 | 0.1093 | 0.087 | 0.6111 | 1 |
|  | RC LP | 0.1039 | 0.0836 | 0.1046 | 0.0876 | 0.9758 | 1 |
|  | RC RP | 0.1038 | 0.0967 | 0.08 | 0.0781 | 0.1792 | 1 |
|  | MP O | 0.1224 | 0.1045 | 0.1333 | 0.1047 | 0.4479 | 1 |
|  | MP LT | 0.1055 | 0.0882 | 0.0845 | 0.0664 | 0.457 | 1 |
|  | MP RT | 0.1016 | 0.0928 | 0.0887 | 0.0539 | 0.6111 | 1 |
|  | MP LP | 0.1107 | 0.099 | 0.0908 | 0.0538 | 0.6875 | 1 |
|  | MP RP | 0.1511 | 0.1498 | 0.1355 | 0.1078 | 0.8913 | 1 |
|  | O LT | 0.0956 | 0.0711 | 0.0866 | 0.0644 | 0.8081 | 1 |
|  | O RT | 0.086 | 0.075 | 0.0802 | 0.0644 | 0.814 | 1 |
|  | O LP | 0.1053 | 0.0939 | 0.1133 | 0.0956 | 0.7327 | 1 |
|  | O RP | 0.1241 | 0.1128 | 0.1271 | 0.1247 | 0.9033 | 1 |
|  | LT RT | 0.1098 | 0.1046 | 0.114 | 0.0782 | 0.3787 | 1 |
|  | LT LP | 0.113 | 0.1033 | 0.0984 | 0.0794 | 0.8674 | 1 |
|  | LT RP | 0.1212 | 0.1053 | 0.0961 | 0.083 | 0.2613 | 1 |
|  | RT LP | 0.0968 | 0.0901 | 0.1001 | 0.0647 | 0.1996 | 1 |
|  | RT RP | 0.1175 | 0.1188 | 0.1051 | 0.0864 | 0.8614 | 1 |
|  | LP RP | 0.1344 | 0.1262 | 0.1236 | 0.1074 | 0.8674 | 1 |
